# EGFR controls *Drosophila* tracheal tube elongation by intracellular trafficking regulation

**DOI:** 10.1371/journal.pgen.1006882

**Published:** 2017-07-05

**Authors:** Ivette Olivares-Castiñeira, Marta Llimargas

**Affiliations:** Developmental Biology Department, Institut de Biologia Molecular de Barcelona, CSIC, Parc Científic de Barcelona, Barcelona, Spain; Harvard Medical School, Howard Hughes Medical Institute, UNITED STATES

## Abstract

Development is governed by a few conserved signalling pathways. Amongst them, the EGFR pathway is used reiteratively for organ and tissue formation, and when dysregulated can lead to cancer and metastasis. Given its relevance, identifying its downstream molecular machinery and understanding how it instructs cellular changes is crucial. Here we approach this issue in the respiratory system of *Drosophila*. We identify a new role for EGFR restricting the elongation of the tracheal Dorsal Trunk. We find that EGFR regulates the apical determinant Crb and the extracellular matrix regulator Serp, two factors previously known to control tube length. EGFR regulates the organisation of endosomes in which Crb and Serp proteins are loaded. Our results are consistent with a role of EGFR in regulating Retromer/WASH recycling routes. Furthermore, we provide new insights into Crb trafficking and recycling during organ formation. Our work connects cell signalling, trafficking mechanisms and morphogenesis and suggests that the regulation of cargo trafficking can be a general outcome of EGFR activation.

## Author summary

The control of organ size and shape is a critical aspect of morphogenesis, as miss-regulation can lead to pathologies and malformations. The tracheal system of *Drosophila* is a good model to investigate this issue as tube size is strictly regulated. In addition, tracheal system development represents also an excellent system to study the molecular mechanisms employed by signalling pathways to instruct cells to form tubular structures. Here we describe that EGFR, which triggers one of the principal conserved pathways acting reiteratively during development and homeostasis, is required to restrict tube elongation. We find that EGFR regulates the accumulation and subcellular localisation of Crumbs and Serpentine, two factors previously known to regulate tube length. We show that Crumbs and Serpentine are loaded in common endosomes, which require EGFR for proper organisation, ensuring delivery of both cargoes to their final destination. We also report that during tracheal development the apical determinant Crumbs undergoes a complex pattern of recycling, which involves internalisation and different sorting pathways. Our analysis identifies EGFR as a hub to coordinate both cell intrinsic properties, namely Crumbs-dependant apical membrane growth, and extrinsic mechanisms, Serpentine-mediated extracellular matrix modifications, which regulate tube elongation. We suggest that the regulation of the endocytic traffic of specific cargoes could be one of the molecular mechanisms downstream of the EGFR, and therefore could regulate different morphogenetic and pathological EGFR-mediated events.

## Introduction

Understanding how organs form and are maintained is a major goal in developmental biology. The *Drosophila* tracheal system is an excellent model to analyse the morphogenesis of branched tubular organs. Tubular structures are present in all organisms and accomplish basic functions of gas and liquid transport [1,2]. Embryonic tracheal development involves a morphogenetic phase where the tubular structure is formed [3]. In parallel, there is a maturation phase where the tubes attain their final size and shape and become physiologically active, able to transport oxygen [4]. The control of tube size and shape is key, as loss of regulation can lead to pathologies and malformations [2,5]. Diametric/circumferential growth of tracheal tubes occurs at stage 14 and correlates with a pulse of secretion that ensures apical membrane growth and secretion of contents into the lumen [4,6]. This secreted luminal material, which includes chitin, chitin-associated proteins and ZP proteins like Pio-Pio and Dumpy, forms an apical extracellular matrix (aECM) that organises into an elastic filament [7,8]. This filament plays an important role in the control of the size and stability of the tracheal tubes [9,10]. Longitudinal/axial growth of tracheal tubes occurs in a continuous manner after diametric expansion. It depends on intrinsic cell properties such as Crumbs-dependant (Crb) apical membrane growth [7,11] and pSrc-dependant polarised cell shape changes [12,13]. In addition, it also depends on extrinsic mechanical forces exerted by the aECM that counteracts and restricts tube elongation. In particular, it was shown that the chitin filament needs to be properly organised and modified by chitin-associated proteins like the chitin deacetylases Vermiform (Verm) and Serpertine (Serp) to prevent excessive growth [14,15]. While we have some hints into how these intrinsic cell properties and the aECM crosstalk and are balanced to control the final tube size [7,16] we still don't have a complete picture of how it works.

Work from different labs has identified roles in tracheal formation of several conserved signalling pathways that regulate development and homeostasis in multicellular organisms. The Epidermal Growth Factor Receptor (EGFR), a Tyrosine Kinase Receptor (RTK), triggers one of these conserved signalling pathways. EGFR is critical during embryonic development controlling important aspects such as migration, differentiation, proliferation, cell growth and survival [17,18]. In addition, miss-regulation of EGFR activity has been associated with cancer and metastasis, and is a target for therapies [19]. EGFR is used in a reiterated manner during the development of different organs in *Drosophila*, such as in the eye (for a review see [20]). Interestingly, EGFR also displays different requirements during tracheal formation, being required at different steps: for invagination [21–24], branching [25,26] and epithelial integrity [27]. Given the relevance of EGFR, it is important to investigate the exact molecular mechanism/s downstream of EGFR that lead to these different outcomes, and understand how they then instruct the cellular changes.

The endocytic pathway uptakes cargoes (membrane proteins, ligands, lipids or extracellular material) and return them back to the membrane or the extracellular space, ensuring their correct recycling or relocalisation. After endocytosis, internalised cargoes arrive to sorting endosomes, from where they are sorted to the final destination through different specific routes. Membrane cargoes destined to degradation will be targeted to intraluminal vesicles (ILV), which will fill the multivesicular bodies, previous to delivery to the lysosome. Alternatively, cargoes can either undergo retrograde transport to the TransGolgi Network (TGN), accessing the secretory pathway, or they can recycle to the plasma membrane using a direct or an indirect (through the recycling endosome, RE) route [28,29]. Each of these trafficking steps in the endocytic pathway is associated and mediated by different Rab proteins. For instance, while Rab5 mediates internalisation and targeting to early endosomes (EE), Rab4 and Rab35 are associated with a short loop recycling pathway back to the plasma membrane [30,31]. Cargo retrieval to the TGN or to the membrane usually relies on the presence of sorting signals in the cargoes that are recognised by coat complexes. This interaction facilitates the partition of cargoes into different discrete domains in the sorting endosome and the subsequent formation of distinct transport intermediates delivered to the final destination. One of these coat complexes is the Retromer complex, which rescues cargoes from degradation using the retrograde transport or direct delivery to plasma membrane. The Retromer associates with different proteins, like sorting nexins, providing specificity for cargo and trafficking itinerary [32–35]. In addition, the Retromer associates with the WASH complex, an actin-polymerisation promoting complex that generates discrete actin patches that facilitate cargo sorting, Retromer tubule formation and fission [34].

Here we identify a new tracheal requirement of EGFR signalling in regulating tracheal axial growth. Downregulation of the pathway leads to overelongated tubes. We find that EGFR acts on the proper accumulation and subcellular localisation of different factors known to participate in the control of tube length, namely the apical determinant Crb and the chitin deacetylase Serp. A detailed analysis of this regulation shows that these two proteins traffic together in common sorting endosomes, from which they will reach their final destination. Serp was shown to undergo retrograde transport using the Rab9-Retromer complex machinery to recycle to the lumen [36]. Here we find that, in the trachea, Crb also undergoes a complex pattern of recycling. Our data indicates that after Rab5-mediated internalisation, Crb accumulates in Serp/Crb endosomes from which a fraction of the internalised protein is targeted to degradation and another fraction is recycled back to the apical domain, using Rab11 and Retromer/Rab4 routes. Dominant negative mutations of EGFR affect the organisation of the Crb/Serp sorting endosomes, likely affecting the correct trafficking of both cargoes. Our results also suggest that Serp and Crb influence each others' recycling, raising the possibility that they are not only cargo but also direct or indirect regulators of endocytic trafficking. Our analysis identifies EGFR signalling as a hub to coordinate both cell intrinsic properties and extrinsic mechanisms that regulate tube elongation. EGFR is a key signalling pathway that participates in many different processes during normal development and pathogenic conditions, and we suggest that the regulation of the endocytic traffic of specific cargoes could be one of the molecular mechanisms used.

## Results

### The modulation of EGFR activity controls tube growth

**Fig 1:**
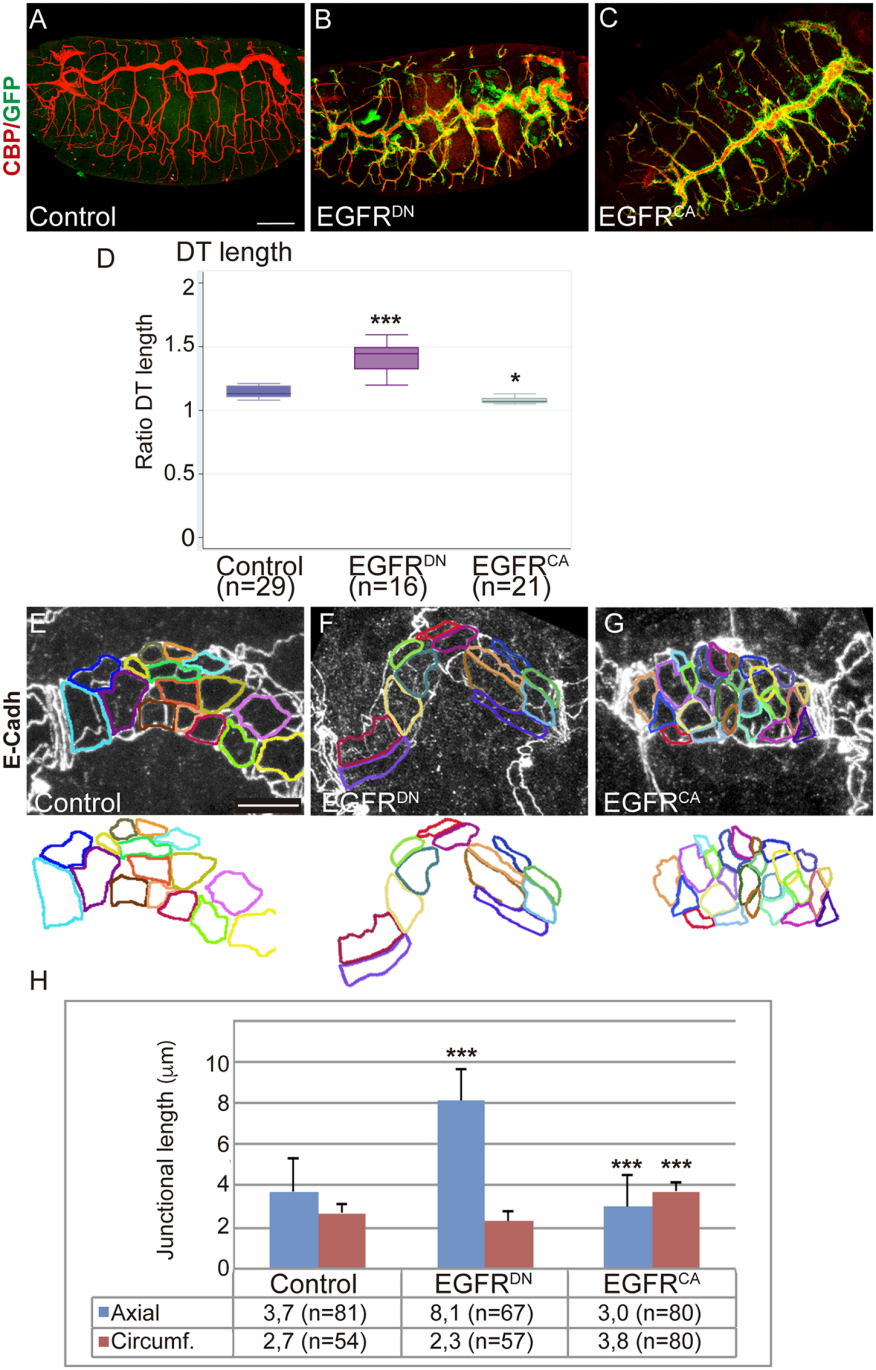
EGFR controls the elongation of the DT. (A-C) Lateral views of stage 16 embryos stained for GFP (green) and CBP (red, lumen).Compare the elongated DT when EGFR is downregulated (*btlGal4-UASsrcGFP-UASEGFR*^*DN*^) to control (*UAS-EGFR*^*DN*^) and to EGFR overactivation (*btlGal4-UASsrcGFP-UASEGFR*^*CA*^). Scale bar 50 μm (D) Box plot for Ratio DT quantification (length of the tube/linear length) of the different experimental conditions of stage 16 embryos. n, number of embryos analysed. The single (*) and triple (***) asterisks represent P<0.05 and P<0.001, respectively, compared with the control by Student's t-test. (E-G) Details of 1 single tracheal metamere of stage 16 embryos stained for E-Cadh to visualise the apical cell shape. The apical outline is highlighted in colours. Note the axial elongation in EGFR^DN^ mutant cells and the circumferential expansion in EGFR^CA^. Scale bar 7,5 μm (H) Quantification of the length of the axial and circumferential junctions. n, number of cells analysed from 3 different embryos per genotype. Triple asterisks (***) represent a significant difference from the control by Student's t-test (P<0.001).

Previous analysis identified roles of the EGFR pathway in regulating invagination and the integrity of the tracheal branches [23,27]. In addition to this requirement, we noticed that at late embryonic stages the size and shape of the tracheal tubes were abnormal when EGFR was downregulated (EGFR^DN^) or constitutively activated (EGFR^CA^) in tracheal cells. In particular, we found a highly convoluted Dorsal Trunk (DT) in EGFR^DN^ mutants and a shorter one in EGFR^CA^ (Fig 1A–1C, S1A–S1C Fig). We quantified this phenotype by measuring the tubes of EGFR^DN^ and EGFR^CA^ mutants at stage 16. We measured the length of the DT, of Dorsal Branch 5 (DB5), of Transverse Connective 5 (TC5), and the diameter of the DT in metamere 7–8. The quantification indicated a clear elongation of the DT when the receptor is downregulated (the DT of EGFR^DN^ is 22,6% longer than the sibling control embryos), and a mild reduction (3%) in EGFR^CA^ (Fig 1D), while the embryo length did not change (S1G Fig). This correlated with a subtle diametrical enlargement of the tubes in EGFR^CA^ embryos (S1F Fig). Length of the rest of the branches was unchanged (S1D and S1E Fig), indicating a specific role of the EGFR in regulating the proportion of the multicellular DT.

To characterise the nature of the elongation we analysed cell number to determine if an increase could cause an enlarged DT in EGFR^DN^ mutants. We found comparable numbers of cells in metamere 7–8 DT region in mutants and control (25,5 cells in control embryos, n = 4 embryos, and 26 cells in EGFR^DN^, n = 3 embryos). We then analysed cell shape of DT cells, as it is documented that cell morphology impinges on tube size and shape [12,13]. We observed that cells were elongated along the anterioposterior axis in EGFR^DN^ mutants (Fig 1F). By contrast, cells in EGFR^CA^ embryos were abnormally oriented diametrically (Fig 1G). We measured the length of the axial cell junctions and of the circumferential junctions in control and mutant embryos. We detected a significant bias towards an enlargement of the axial junctions in EGFR^DN^ and a significant bias towards a reduction in EGFR^CA^ (Fig 1H). Modulation of the activity of Breathless, Btl, another RTK with critical roles in tracheal formation (reviewed in [3]), did not affect tube size and cell shape (S1H–S1K Fig), indicating that EGFR effects were specific.

Altoghether these results indicate that the EGFR activity prevents the excessive DT elongation and regulates the morphology of DT cells.

### EGFR controls Serp accumulation in the aECM

During embryonic development the lumen of tracheal tubes is filled transiently with an aECM composed of chitin and chitin associated proteins. This matrix plays a key role in controlling tube size and shape [9,10]. The chitin binding protein Serp specifically restricts excessive tube elongation by regulating the structural properties of this chitin filament [14,15]. Hence, in the absence of Serp the tracheal tubes overelongate, showing a phenotype similar to that of the EGFR downregulation. This prompted us to analyse a possible relationship between EGFR and Serp.

**Fig 2:**
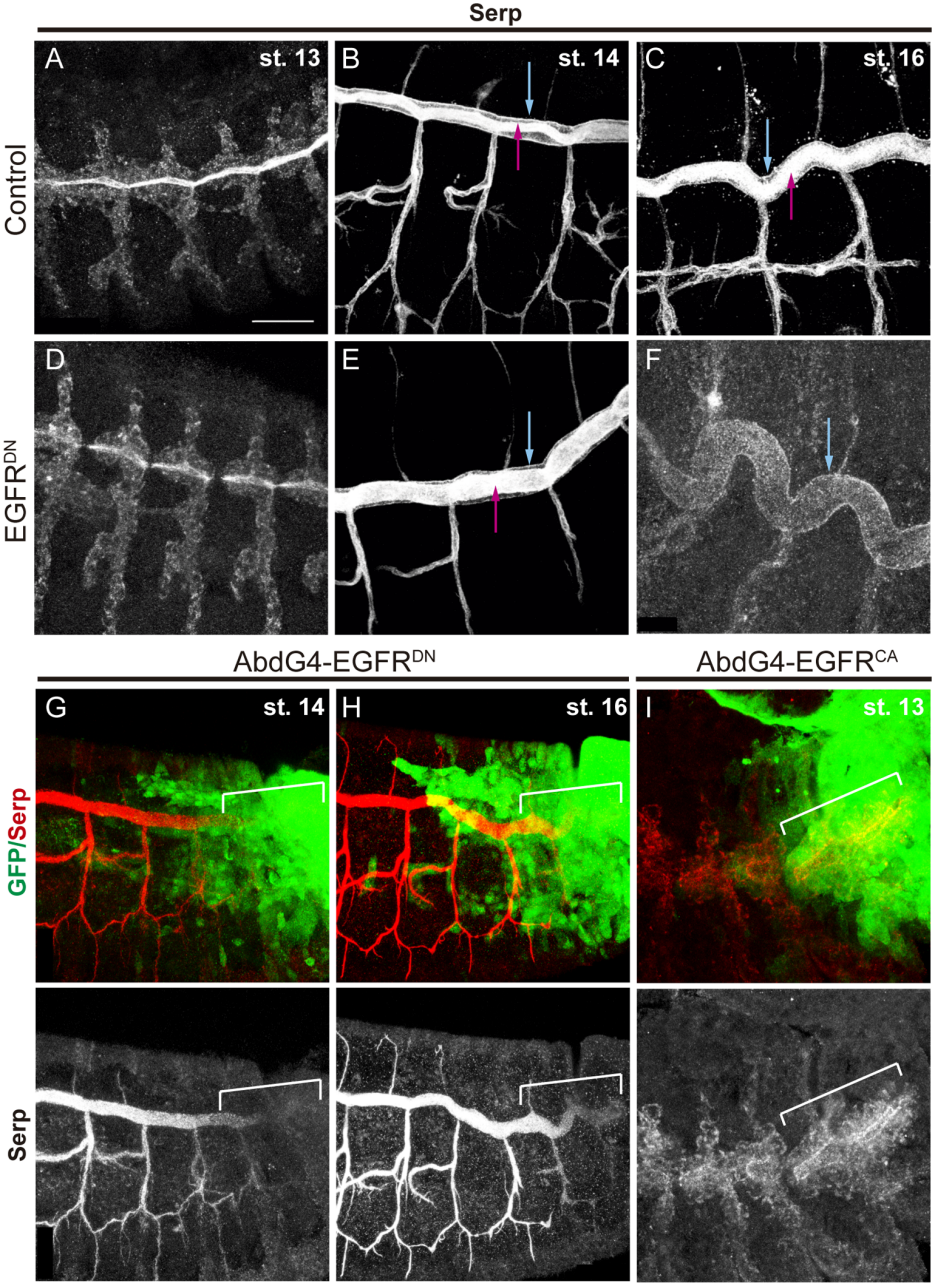
EGFR regulates the luminal accumulation of Serp. (A-F) Lateral views of embryos stained with Serp antibody at the indicated stages. From early stages Serp can be detected in the lumen (pink arrows) and in the apical membrane of tracheal cells (blue arrows). When EGFR is downregulated, Serp accumulation in the lumen decreases strongly with time, but remains detectable in the apical membrane (blue arrow in F). (G-I) Lateral views of embryos at the indicated stages carrying *AbdGal4-UASGFP* stained with Serp antibody (red or white) and GFP (green). Note the difference of Serp luminal accumulation in the Abd domain (marked by a white bracket) in the different EGFR mutant conditions. Scale bar 25 μm.

Serp is first detected in the cytoplasm of tracheal cells at stage 13. From stage 14, and until stage 16, Serp is secreted and accumulates in the lumen of the tubes (Fig 2A–2C; S2A–S2C Fig, pink arrows), colocalising with the chitin filament [14]. In addition, Serp also accumulates from early stages in the apical membrane of the tracheal cells, lining the lumen (Fig 2B and 2C; S2B and S2C Fig blue arrows). We analysed Serp accumulation when EGFR^DN^ was expressed in the trachea. Serp was detected in the tracheal cells at early stages (Fig 2D) and in the apical membrane (Fig 2E and 2F blue arrowhead), as in the control. However, we detected a decrease of Serp accumulation in the lumen at late stages (Fig 2F). This was especially clear when we expressed EGFR^DN^ in the posterior part of the tracheal system (Fig 2G and 2H).

The expression of EGFR^CA^ has a mild effect on Serp accumulation, which seems to start accumulating in the lumen slightly earlier than in the control, while at later stages this luminal accumulation remains strong (Fig 2I; S2D–S2F Fig).

As Serp has been shown to control tube elongation and we find that EGFR controls Serp luminal accumulation, we propose that EGFR controls tube size at least in part by regulating Serp accumulation.

### EGFR controls Crb accumulation in the DT

Besides the key role of the aECM, the activity of *crb* also correlates with tube elongation. Overexpression of *crb* [11] or an increased *crb* activity [7] leads to an overproduction of apical membrane that results in overelongated tubes. In line with these reports, we previously showed that Crb levels are downregulated at late stages [37], consistent with the idea that Crb is finely regulated to prevent excessive growth.

**Fig 3:**
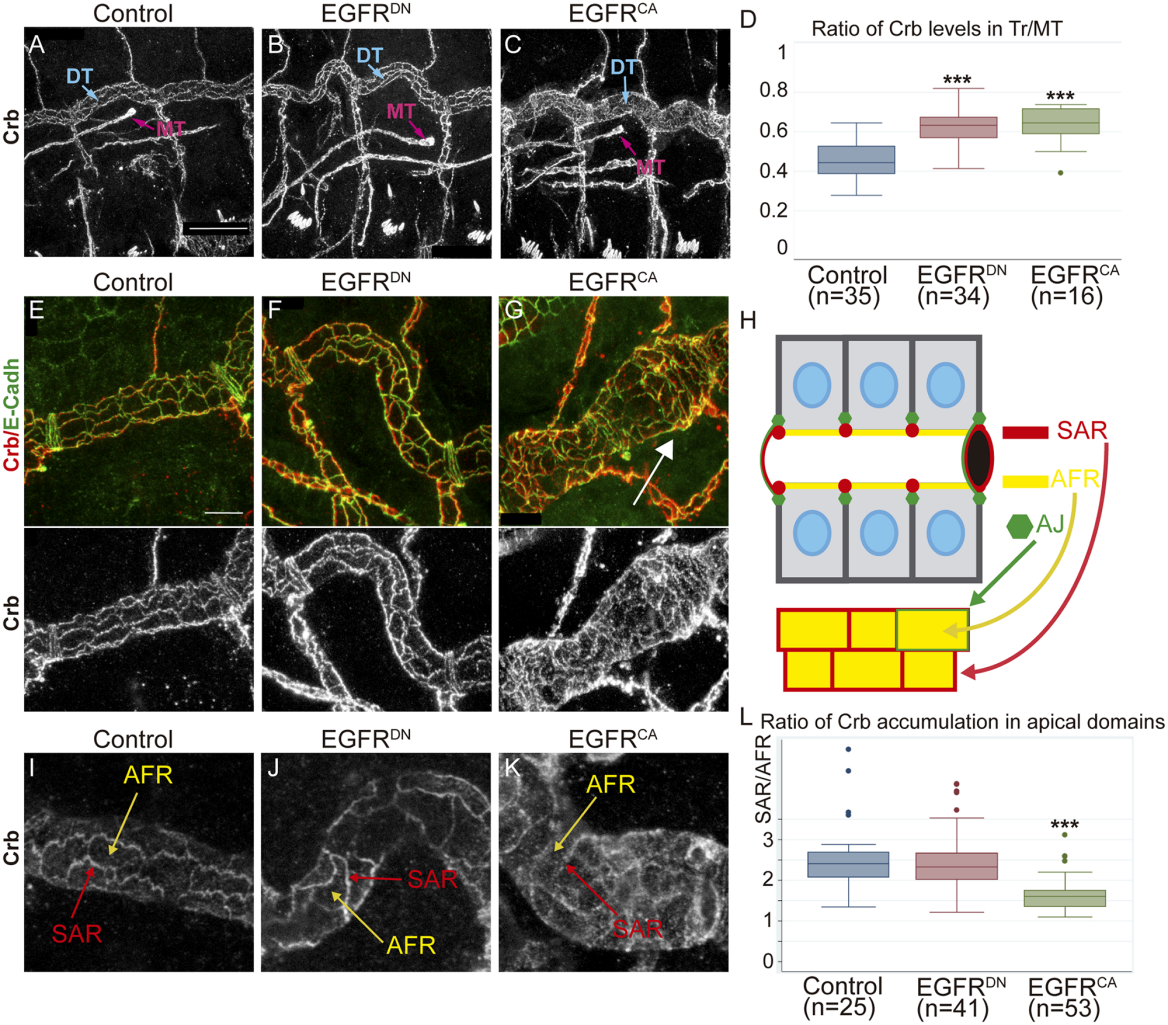
EGFR regulates the accumulation of Crb. (A-C) Lateral views of stage 16 embryos of indicated genotypes stained for Crb. Panels show the DT of 3 tracheal metameres and a MT to compare Crb levels. MT do not express EGFR constructs. Scale Bar 25 μm (D) Quantification of Crb levels. The ratio significantly increases in EGFR mutants, indicating higher levels of Crb in the DT. n, number of embryos analysed. Triple asterisks (***) represent a significant difference from the control by Student's t-test (P<0.001) (E-G) Details of 1 single tracheal metamere of stage 16 embryos of indicated genotypes stained for E-Cadh (green) and Crb (red, white). Scale bar 7,5 μm (H) Diagram representing a fragment of the DT in a sagital and a 2D-planar apical view. The apical region of the tracheal cells faces the lumen of the tube (black hole). Crb accumulates in the apical free region (AFR, yellow), in direct contact with the lumen, and in the Subapical region (SAR, red), contacting two adjacent cells. E-Cadh accumulates basal to the SAR in AJs (green) (I-K) Details of 1 single tracheal metamere of stage 16 embryos of indicated genotypes stained for Crb. Panels show an example used to evaluate Crb subcellular accumulation in the AFR (yellow arrows) and the SAR (red arrows). (L) Quantification of the ratio of Crb accumulation in apical region. Note that when EGFR is overactivated Crb enrichment in the SAR is decreased. n, number of DT cells analysed from 5 control, 9 EGFR^DN^ and 11 EGFR^CA^ embryos. Triple asterisks (***) represent a significant difference from the control by Student's t-test (P<0.001).

Hence, we analysed Crb accumulation in EGFR mutant conditions. In embryos expressing EGFR^DN^ in the trachea, Crb normally localised apically throughout all tracheal development. However, we detected a bright and sharp accumulation of Crb compared to the control (Fig 3A and 3B; S3A–S3H Fig). To verify this observation we quantified the levels of Crb at stage 16 in the DT and normalised the value to the levels in Malphigian Tubules (MT) for each embryo (see Materials and Methods). We found that in EGFR^DN^ embryos the levels of Crb in the trachea are higher than in control sibling embryos (Fig 3D). When we quantified Crb levels in EGFR^CA^ conditions we also detected higher levels of Crb as compared to wild type (Fig 3C and 3D; S3I–S3L Fig).

In spite of the apparently similar phenotype of increased Crb levels in EGFR^DN^ and EGFR^CA^, a closer evaluation indicated differences. In stage 16 control embryos we found Crb sharply localised and defined in the Subapical Region (SAR). The SAR corresponds to the most apicolateral region of the membrane, connecting adjacent epithelial cells (Fig 3H, red). In addition, we also detected some Crb accumulated in the most apical membrane, that we name Apical Free Region (AFR), which is in direct contact with the tracheal lumen (Fig 3H, yellow). Accumulation of Crb in the SAR outlines the cell contour, as it does the accumulation of E-Cadherin (E-Cadh), which accumulates in the Adherens Junctions (AJ) (Fig 3E and 3H, green), positioned just below the SAR. In EGFR^DN^ mutant conditions we found Crb well defined in the SAR (Fig 3F; S3N Fig). In contrast, in EGFR^CA^ mutant conditions, the accumulation of Crb in the SAR appeared blurred and faint, while Crb seemed very conspicuous in the AFR (Fig 3G; S3O Fig). This abnormal accumulation was particularly conspicuous in certain regions of the DT, randomly distributed but more frequent in the posterior region. These regions with more delocalised Crb accumulation in the SAR correlated with cell surfaces elongated circumferentially and with a circumferential enlargement of the tube (arrow in Fig 3G). To evaluate this observation we quantified the ratio of Crb levels in SAR versus AFR in individual cells of control and mutant EGFR conditions. The analysis indicated that while the ratio was not very different from the control in EGFR^DN^ mutants (suggesting that Crb is increased in all apical regions), it was significantly different in EGFR^CA^, with less enrichment of Crb in the SAR (Fig 3L).

Altogether our results indicate that EGFR activity regulates Crb levels in the DT. Because Crb has been shown to promote apical membrane growth at late stages of tracheal formation, we propose that EGFR triggers its function through the regulation of Crb levels. In addition, our results also show that Crb can accumulate in different subcellular domains in the apical membrane of tracheal cells.

### Crb subcellular accumulation depends on its endocytic trafficking

**Fig 4:**
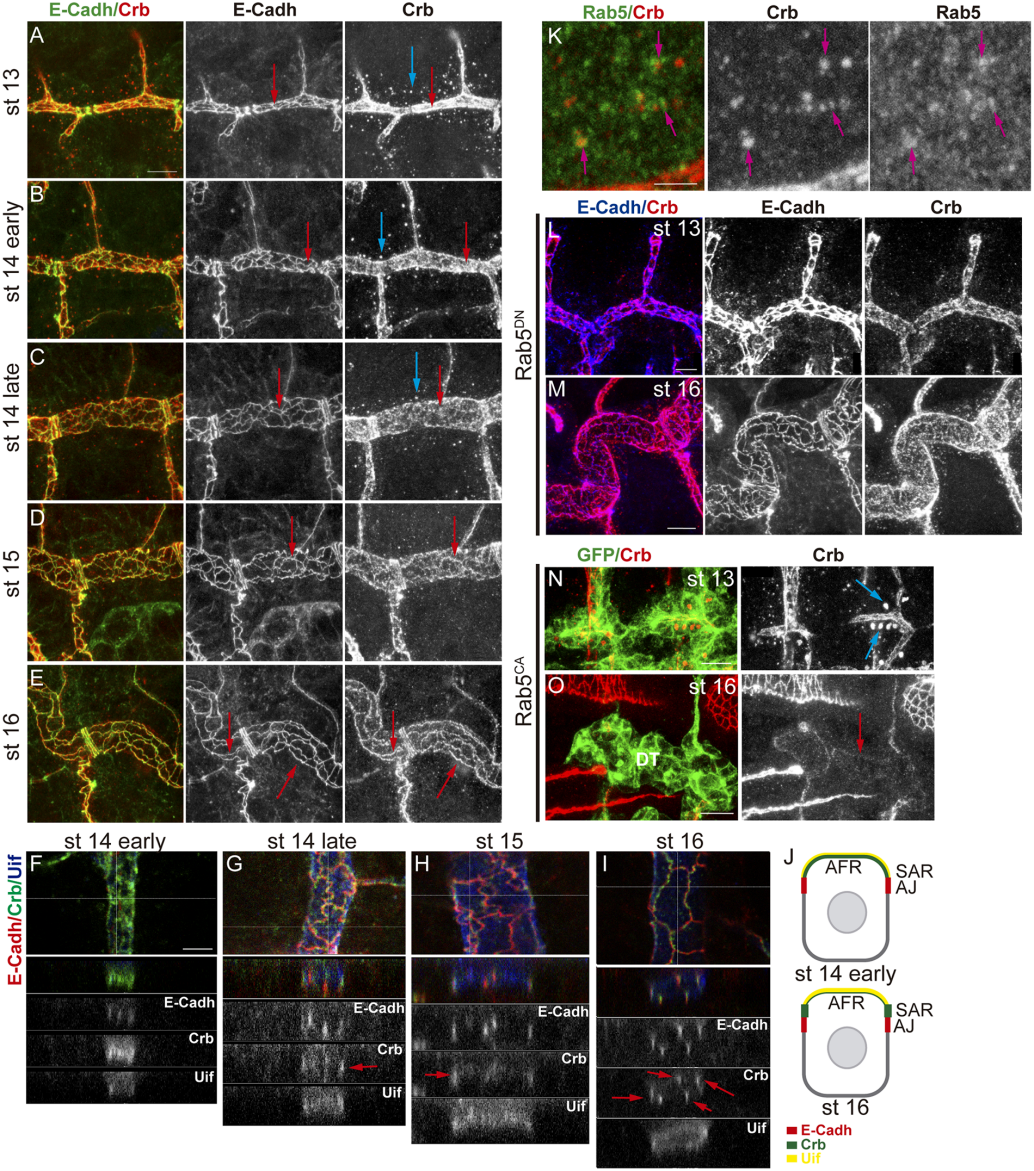
Subcellular accumulation of Crb during tracheal development. (A-E) Lateral views of wild type embryos at indicated stages stained for E-Cadh (green, white) and Crb (red, white), showing 1–2 tracheal metameres. At early stages, while E-Cadh is already detectable in the junctional area (red arrows), Crb mostly accumulates in the AFR. Crb accumulation becomes enriched in the SAR as development proceeds (E). At early stages, abundant vesicles containing Crb are detected (blue arrows), and they decrease as development proceeds. Scale bar 7,5 μm (F-I) Z-reconstructions of the DT of embryos at the indicated stages stained for E-Cadh (red, white), Crb (green, white) and Uif (blue, white). Horizontal line in the upper panel indicates the position of the Z- reconstruction. E-Cadh always localise to the AJs, in the apicolateral membrane, visualised as lines spanning the AJs. Uif localises in the most apical membrane. Note the evolution of Crb pattern that becomes more enriched in the SAR (red arrows, partially colocalising with E-Cadh) as development proceeds. Scale bar 5 μm (J) Diagram representing a Z-section of a DT cell at early and late stages. The apical region of the tracheal cells facing the lumen (AFR) accumulates Uif (yellow). E-Cadh accumulates in AJs (red). Crb (green) first accumulates more in the Uif region and later becomes enriched in the SAR. (K) Wild type embryo stained with Rab5 (green, white) and Crb (red, white) antibodies. Many Crb vesicles co-stain with Rab5 (pink arrows). Scale bar 2,5 μm (L,M) Lateral views of embryos with downregulated Rab5 activity at the indicated stages stained for E-Cadh (blue, white) and Crb (red, white) showing 1–2 tracheal metameres. At early stages Crb vesicles are absent (G), and at late stages (H) Crb is not enriched in the SAR. Scale bar G 5 μm, H 7,5 μm (N,O) Lateral views of embryos with activated Rab5 activity at the indicated stages stained for GFP (green) and Crb (red, white) showing 2 tracheal metameres. At early stages huge Crb vesicles are detected (blue arrows in I), but at late stages (J) Crb is almost absent mainly from the DT region. Scale bar I 7,5 μm, J 10 μm.

Our results suggested that Crb can localise in different apical domains in a regulated manner. We further investigated this subcellular accumulation of Crb at different stages. We used the pattern of E-Cadh, which reveals the cell outline homogeneously throughout all tracheal development (Figs 3H and 4A–4E), to compare to Crb. We observed that at stages 13–14 Crb is mainly localised in the AFR region, with not much enrichment in the SAR (Fig 4A and 4B). However, as development proceeds we detected a progressive enrichment of Crb in the SAR (Fig 4C and 4D; S1 Video). By st 16–17 Crb appears neatly accumulated and can be perfectly distinguished in the SAR (Fig 4E). This evolution of Crb subcellular accumulation in the apical region, which can also be observed in Z-sections (Fig 4F–4J), correlated with a stage specific accumulation of Crb in intracellular vesicles. These Crb vesicles were abundant and clearly detected at stages 13–14 (Fig 4A, 4B and 4C blue arrows), and decreased by late st 14 or st 15, being less frequent at st 16 (Fig 4D and 4E). The inverse correlation between the presence of Crb vesicles prior to its enrichment in the SAR raised the possibility that these two events are related.

We investigated the nature of these vesicles. Diameter quantification indicated that they are around 0,5–0,6 μm (n = 21 vesicles, from 3 different embryos), consistent with being endosomes [38]. To determine their nature we double stained for different Rab proteins, which identify different intracellular trafficking vesicles [30,31]. Rab5 mediates traffic from the plasma membrane to EE and serves as marker for EE. We found that the Crb vesicles are rich in Rab5, further suggesting that these are endosomes (Fig 4K). To confirm this we expressed a dominant negative form of Rab5 in the trachea to block endocytosis. We found that the 0,5–0,6 μm Crb vesicles typically found at stages 13–14 disappeared (Fig 4L; S4A Fig). Interestingly, we also found that when endocytosis was blocked, Crb remained high in the AFR and did not become enriched in the SAR at late stages (Fig 4M), suggesting a dynamic recycling of Crb at different apical domains dependent on internalisation. The expression of a constitutively active Rab5 protein induced the formation of enormous Crb endosomes at early stages (Fig 4N). At later stages, Crb was almost absent from the trachea, particularly from the DT (Fig 4O), suggesting that most Crb accumulating in the big endosomes is targeted to degradation.

Altogether the results indicate that Crb undergoes a highly dynamic pattern of subcellular localisation throughout tracheal development, refining at the SAR at late stages. This pattern requires Rab5-dependent endocytosis.

### Crb apical localisation and recycling pathways

Our results suggested that during tracheal development Crb is endocytosed and likely recycled back to the SAR. It has been shown in other tissues that different endosomal sorting pathways control Crb trafficking regulating its activity. While a fraction of the pool of internalised Crb protein undergoes degradation [8], Crb is also recycled back to the apical membrane through Rab11/ [39] Exo84 [40] or the Retromer complex [41,42] dependent pathways. We asked whether any of these pathways is required for Crb trafficking and recycling during tracheal development.

**Fig 5:**
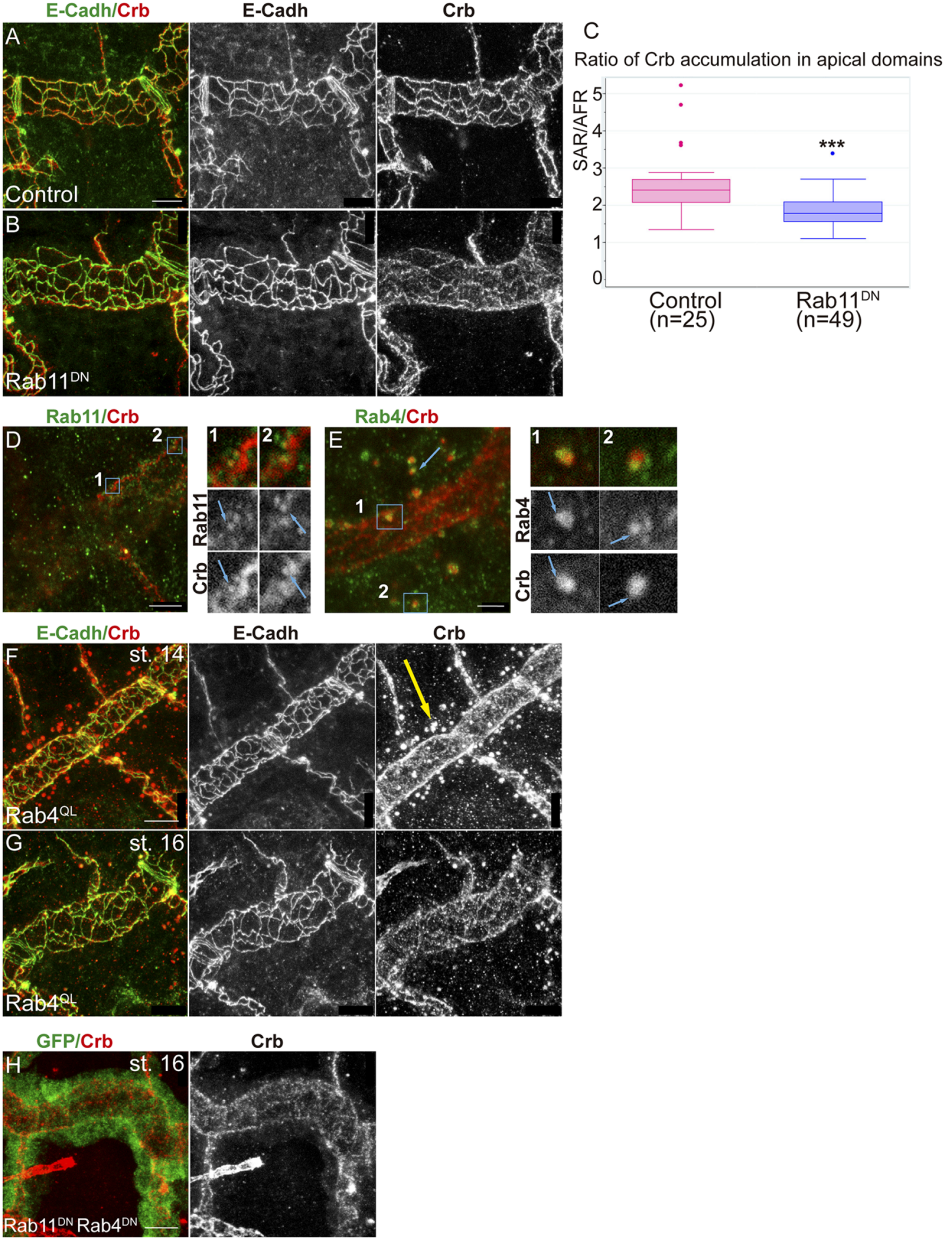
Trafficking of Crb through the endocytic pathway. (A,B) Lateral views of stage 16 embryos stained for Crb (red, white) and E-Cadh (green, white) showing 1 tracheal metamere. Note the pattern of accumulation of Crb when Rab11 is downregulated in the trachea. Scale bars 7,5 μm (C) Quantification of the ratio of accumulation of Crb in different apical regions. When Rab11 is downregulated Crb enrichment in the SAR is decreased. n, number of DT cells analysed from 5 control and 11 Rab11^DN^ embryos. Triple asterisks (***) represent a significant difference from the control by Student's t-test (P<0.001) (D,E) Single confocal sections to show the colocalisation of Crb and Rab11 (using anti-Rab11, D) or Rab4 (using Rab4^EYFP^, E). Panels marked with 1 and 2 correspond to the insets shown in D and E. (D) Note the presence of small Crb vesicles, in contact with the junctional area, marked with Rab11 at late stages (stage 15, blue arrows in 1 and 2). (E) Most endosomes present at early stages containing Crb are marked with Rab4. Rab4 and Crb colocalise in regions of these endosomes (blue arrows in 1 and 2). Scale bar D 5 μm, E 2,5 μm (F-H) Lateral views of embryos expressing the indicated transgenes in the trachea stained for Crb (red, white) and E-Cadh (green, white) (except H, stained for GFP, green), showing 1–2 tracheal metameres. Note that Crb accumulates in big endosomes at early stages when Rab4 is constitutively activated (yellow arrow in F), and decreases at later stages (G) or when both Rab11 and Rab4 are downregulated (H). Scale bars 7,5 μm.

We analysed the contribution of the RE-Rab11 dependent trafficking on Crb accumulation. We found that the downregulation of Rab11 activity (expressing a dominant negative construct in the trachea) produced defects in Crb accumulation. At early stages we did not detect defects and Crb was enriched in the AFR and found in endosomes as in the wild type (S4B and S4C Fig). However, at late stages Crb was not specially enriched in the SAR as much as it is in normal conditions, whereas E-Cadh was still detected in the junctional area (Fig 5A and 5B). We quantified the ratio of Crb accumulation in the SAR versus the AFR and found it to be significantly different from the control, being biased towards a depletion in the SAR (Fig 5C). This result suggested a role for the RE route for Crb recycling in the trachea. In agreement with this we detected Rab11-REs containing Crb. These were not the endosomes detected at early stages, as they were smaller (0,28 μm, n = 6 recycling endosomes from 2 different embryos), detected at later stages, and typically close to cell junctions (Fig 5D). Altogether these results are consistent with a role of Rab11 in recycling Crb, particularly to the SAR junctional area.

To further characterise the abundant Crb endosomes present at early stages we used tagged Rab proteins. We found that these endosomes were rich in Rab4. A close look showed that Rab4-YFP-tagged protein decorated the Crb endosomes (Fig 5E, S2 Video), suggesting that a pool of Crb protein accumulates in the lumen of the endosome, likely to be directed to degradation. We also detected some puncta of Crb/Rab4 colocalisation, suggesting a Rab4-mediated recycling (Fig 5E1 and 5E2). It has been shown that Rab4 participates in a short loop endocytic pathway to recycle cargoes back to the membrane [30], and that it can associate with Retromer tubules [43]. The Retromer complex has been shown to participate in Crb recycling [41,42]. In light of this evidence, our results suggested that Crb could recycle using a Rab4/Retromer-dependent pathway. In agreement with this hypothesis we found that when Rab4 is constitutively active, bigger vesicles containing Crb are detected, and they are present until late embryonic stages (Fig 5F, yellow arrow). This correlates with a poorer enrichment of Crb in the SAR at late stages (Fig 5G), suggesting a hypothetical route of Crb recycling mainly to the AFR involving Rab4/Retromer. Interestingly, in *EGFR*^*DN*^ mutants we detected an advanced enrichment of Crb in the SAR as compared to control (S5 Fig). A dominant negative form of Rab4 expressed in the trachea did not produce clear defects (S4D and S4F Fig).

Altogether our results are consistent with a model where, after Rab5-mediated internalisation, a pool of Crb would be retrieved from the degradation pathway to undertake different recycling routes. One of them would involve the Rab4/Retromer complex and would ensure the fast recycling directly to the apical, preferentially the AFR. A slower/longer recycling route, involving Rab11-RE, would traffic Crb from the endosome to the apical membrane, mainly in the SAR. In agreement with a role of both routes in Crb recycling we found that downregulating both of them leads to a strong decrease of Crb in apical regions, particularly in the DT (Fig 5H).

### Serp and Crb localise in common sorting endosomes

Serp has been shown to undergo a specific recycling that maintains its luminal content, which is necessary to restrict tube elongation. Serp recycling requires Rab9, the Retromer complex and WASH to mediate its retrograde trafficking to the lumen through the TGN [36]. Similarly, here we describe that Crb trafficking is also regulated to maintain its apical localisation. We analysed the relative subcellular localisation of the two proteins in tracheal cells.

**Fig 6:**
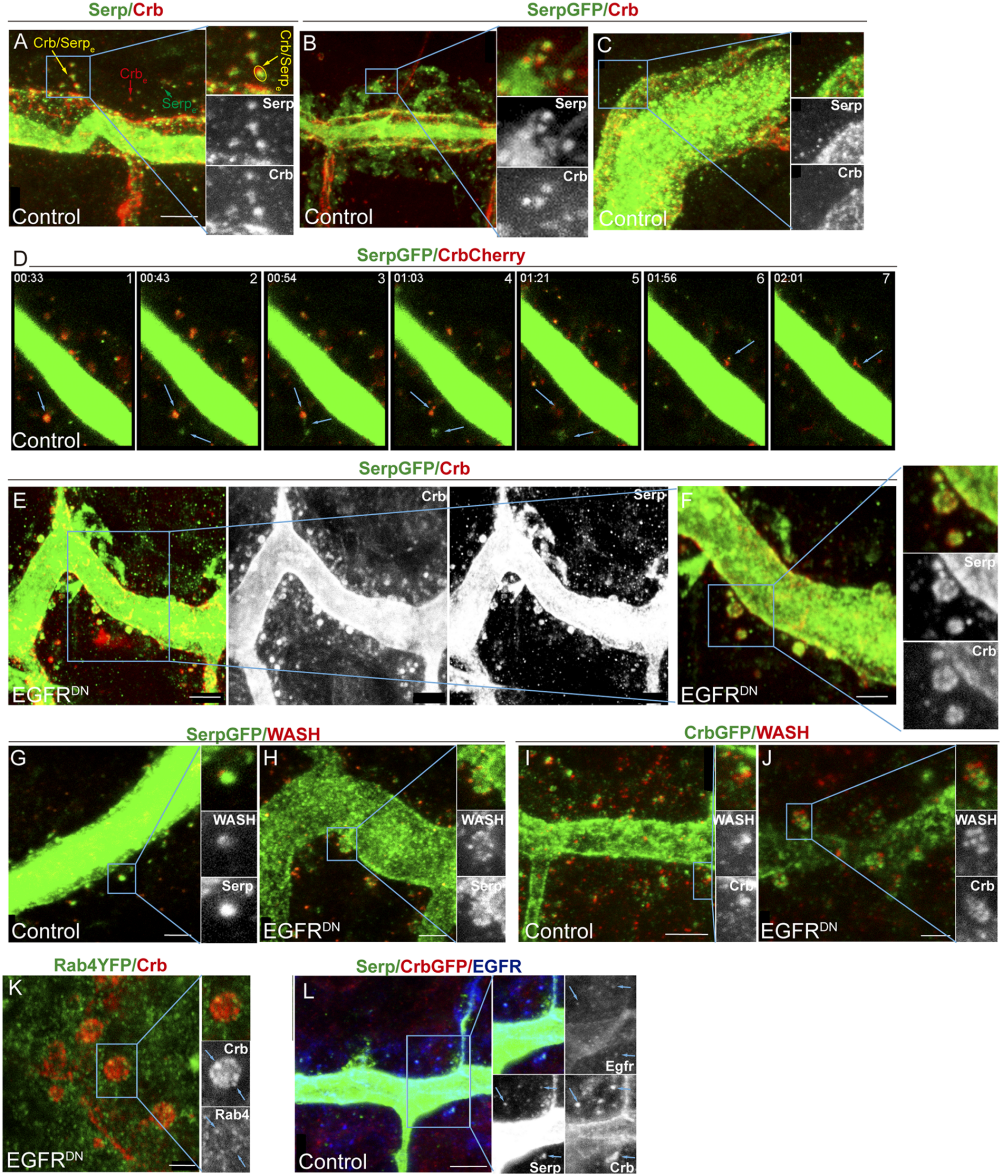
Crb and Serp accumulate in common endosomes, which require EGFR activity. (A-C) Lateral views of WT (A) or *btlGal4-UASSerp-CBD-GFP* (B,C) embryos stained for Crb (red, white) and Serp/GFP (green, white). At stage 14 (A,B) many endosomes accumulate both Serp and Crb (Crb/Serp_e_), but endosomes containing only Serp or Crb (Serp_e_ and Crb_e_, respectively) are also found. In Crb/Serp_e_ the two proteins are sorted into different endosomal domains (insets) but they colocalise in a region. At stage 16 (C) Serp endosomes are still abundant. Scale bar 5 μm (D) Time-lapse images of tracheal cells carrying *btlGal4-UASSerp-CBD-GFP* and *Crb*^*Cherry*^. Crb/Serp endosomes are highly dynamic. Bottom arrows point to an endosome from which Serp is sorted, joining another Serp containing vesicle (2–5). A fraction of Crb protein is also sorted and the rest fades (4–5). Upper arrows in 6–7 point to a Crb tubulation. (E-F) Lateral views of stage 14 embryos carrying *btlGal4-UASSerp-CBD-GFP* and EGFR^DN^ stained for GFP (green, white) and Crb (red, white). Endosomes are bigger and Serp and Crb are abnormally localised, missorted and intermingled. The cargoes often accumulate at the endosome surface (insets in F). Scale bar E 5 μm, F 2,5 μm (G-J) Lateral views of stage 14 embryos carrying *btlGal4-UASSerp-CBD-GFP* (G,H) or Crb^GFP^ (I,J) stained for GFP (green, white) and WASH (red, white). In control embryos typically one or two punctae are detected per Crb/Serp endosome. In *EGFR*^*DN*^ embryos WASH accumulates in more punctae forming a rosette around the endosome. Scale bar G,H,J 2,5 μm, I 5μm (K) Lateral view of a stage 14 embryo expressing EGFR^DN^ in the trachea and carrying Rab4^EYFP^ to visualise Rab4 accumulation. Rab4 is less nicely accumulated and frequent Crb/Rab4 punctae are detected (arrows in inset). Scale bar 2,5 μm (L) Lateral view of a stage 14 embryo carrying Crb^GFP^ and stained for GFP (red, white), Serp (green, white) and EGFR (blue, white). EGFR itself is found very often in Serp/Crb containing endosomes (blue arrows). Scale bar 5μm.

We found very often that Crb and Serp partially co-localise, indicating that they localise in a common endosome. This common localisation increased as development proceeds and reached a peak at stage 14 (Fig 6A and 6B). Quantifications in embryos at stage 14 indicated that 66% of vesicles contained both Crb and Serp, 15% contained only Crb and 18% contained only Serp (Crb/Serp_e_, Crb_e_ and Serp_e_, respectively, in Fig 6A. n = 194 vesicles from 5 different embryos; S6A–6C' and S6D Fig). From stage 15 onwards Crb endosomes decreased or became smaller while Serp endosomes were maintained (Fig 6C). As mentioned, Serp and Crb do not perfectly colocalise but rather they seem sorted into different regions of a common endosome (insets in Fig 6A and 6B and yellow arrow in A; S6A'–S6C' and S6D Fig). Image analysis and co-localisation quantification indicated around a 60% of co-localisation of Serp/Crb signal in common sorting endosomes (n = 118 endosomes, from 5 different embryos). This result indicates that Crb and Serp are partitioned into different discrete domains in a common sorting endosome.

Live imaging using Serp-GFP and Crb^Cherry^ showed a highly dynamic pattern of vesicle trafficking (Fig 6D; S6G–S6I Fig and S3 and S4 Videos). We could clearly detect endosomes containing both Crb and Serp, very often sorted in different regions. We could observe examples where Crb/Serp endosomes fused or evolved rendering Serp and Crb distinct vesicles (arrows in Fig 6D1–6D4), examples where Crb endosomes seemed to disapear (likely reflecting protein degradation, arrows in Fig 6D4 and 6D5), or examples where Crb or Serp localised in (distinct) tubulations or smaller vesicles (likely reflecting retrieval for recycling, arrow in Fig 6D7).

These results show that Crb and Serp traffic together in common sorting endosomes and are sorted into different domains, consistent with the hypothesis that they use different retrieval pathways to recycle to their final destination.

### EGFR is required for the proper organisation of Serp-Crb endosomes

Because EGFR regulates both Crb apical localisation and Serp luminal accumulation, and because both Crb and Serp accumulation depend on their recycling and localise in common sorting endosomes, we investigated the requirement of EGFR in their trafficking.

In EGFR^DN^ conditions Crb/Serp endosomes were still found, indicating that internalisation was not impaired. This was confirmed by a live endocytosis assay [4] that showed presence of internalised rhodamine-labelled dextran in control and EGFR mutant conditions (S7 Fig). However, Crb/Serp endosomes displayed an abnormal aspect (Fig 6E and 6F; S6E and S6F Fig). Endosomes were bigger in general, measuring around 1,3 μm in diametre (n = 27 endosomes, from 6 different embryos). In the control Crb and Serp normally accumulate in a well-defined region in the endosome, usually differently sorted (Fig 6A and 6B). In EGFR^DN^ conditions their accumulation was more diffuse and both proteins were found intermingled, although generally still differently sorted. Crb and Serp were often detected at the surface of the endosome, rather than in the lumen (Fig 6E and 6F), suggesting that more protein is retrieved from the degradation pathway. This would be consistent with the fact that we detect higher levels of Crb (Fig 3D). Hence, our results show that impairing EGFR activity results in the missorting of both cargoes, consistent with the hypothesis that EGFR is required for the proper sorting of cargoes, particularly Crb and Serp, in endosomal membranes.

The Retromer plays a key role in Serp and Crb recycling. We asked whether EGFR somehow controls Retromer activity. The Retromer recruits the actin nucleator WASH complex, which generates discrete actin patches in the endosomes. We analysed WASH accumulation with respect Crb and Serp in normal and EGFR mutant conditions. We found one or occasionally two WASH punctae partially colocalising with Serp and Crb in wild type embryos (Fig 6G and 6I). In EGFR^DN^ mutant conditions the pattern of WASH accumulation was clearly different. We detected many more small punctae of WASH in the endosomes, often forming a rosette, with Serp or Crb abnormally sorted and intermingled (Fig 6H and 6J). This suggests a role for the EGFR in regulating WASH dynamics on the endosome. We also analysed the accumulation of other markers of the Serp/Crb endosomes, like Rab4. We found that Rab4 was not nicely and strongly decorating Crb endosomes as in the control (Fig 5E). Instead Rab4 accumulation was weaker and diffuse, and we could detect punctae of Crb/Rab4 colocalisation more frequently than in control embryos (Fig 6K). Altogether our results indicate that during tracheal development, EGFR is required to organise the sorting endosome containing Serp and Crb, likely regulating their proper sorting.

Interestingly, we found that EGFR itself also traffics in the same sorting endosomes containing Serp and Crb (Fig 6L, S6J–S6L Fig). Image analysis indicated a 40–50% of co-localisation of EGFR/Serp/Crb or EGFR/Crb or EGFR/Serp signal in common endosomes (n = 109 endosomes, from 7 different embryos). This result raises the possibility that EGFR directly influences the traffic of endosomes in which the receptor is loaded.

### Serp and Crb recycling mutually influence each other

Because Crb and Serp localise in common sorting endosomes, and because their recycling is regulated by common mechanisms, we wondered whether the recycling of one of the cargoes could influence the other.

**Fig 7:**
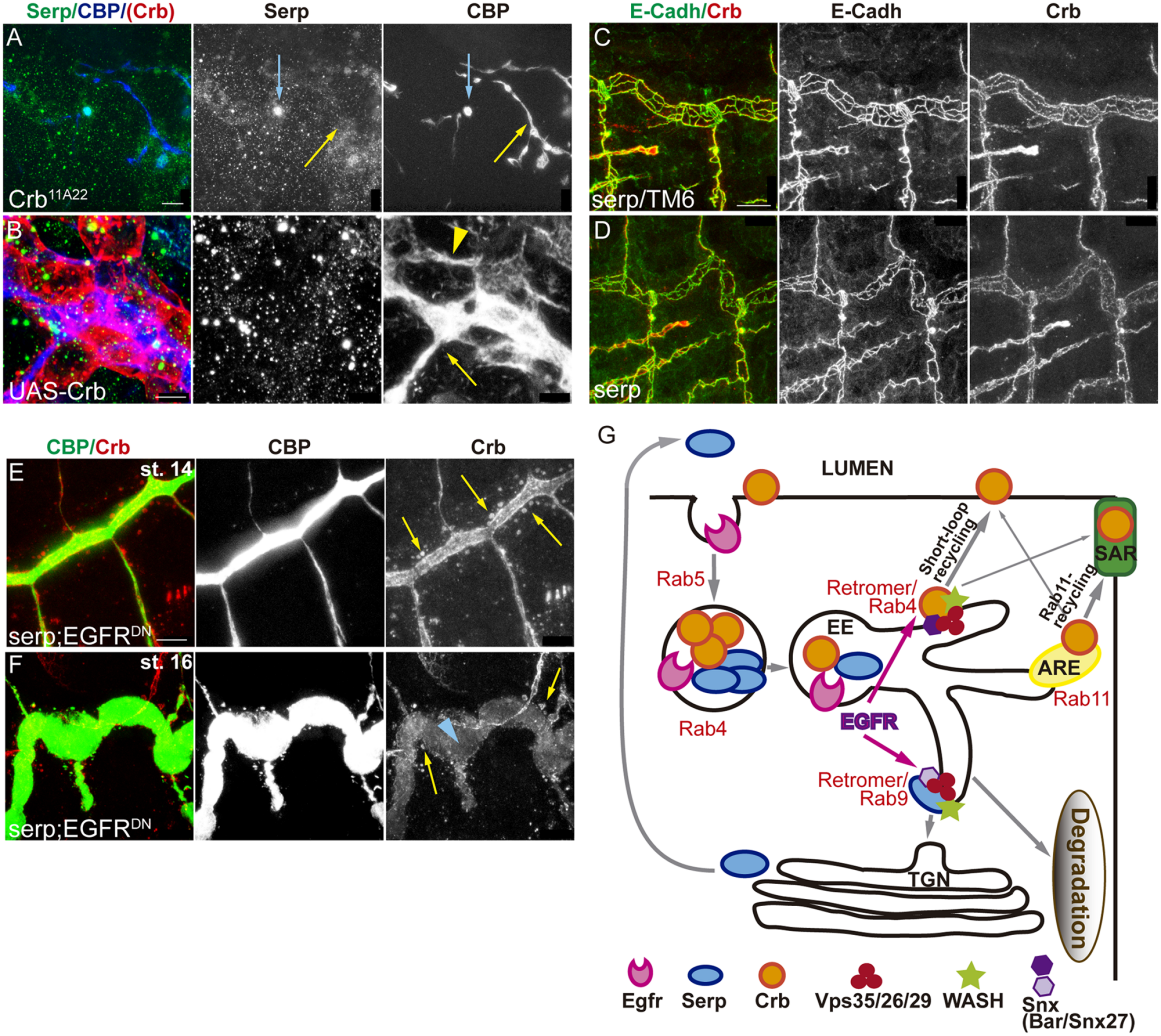
Crb and Serp require each other for proper recycling. (A,B) Lateral views of embryos lacking (A) or overexpressing *crb* (B). (A) In spite of *crb* absence, chitin (visualised with CBP) is deposited in the malformed lumen (yellow arrow), but Serp is only occasionally detected there (blue arrow). (B) When *crb* is overexpressed in trachea it accumulates around the cells, indicating apicalisation. Chitin is deposited extracellularly, in the apical (yellow arrow) and the basal surfaces (yellow arrowhead) but Serp is completely absent. Scale bar A 10 μm, B 5μm (C,D) Lateral views showing 2 tracheal metameres of stage 16 embryos stained for Crb (red, white) and E-Cadh (green, white). Note that Crb staining in the SAR is slightly compromised when *serp* is absent (D), compared to control (C). Scale bar C 10 μm (E,F) Lateral views showing a portion of the DT when *serp* is absent and *EGFR* is downregulated. Note the presence of large Crb vesicles (yellow arrows) at all stages and the depletion of Crb in the apical domain of late embryos (blue arrowhead). Scale bar E 7,5 μm (G) Model. Crb and Serp are endocytosed and arrive to common endosomes, sorted into different domains. A fraction of Crb and Serp internalised protein will be retrieved from the degradation pathway and will be recycled back to the membrane (Crb) or to the lumen (Serp). Serp undergoes retrograde transport using the Rab9-Retromer. Crb uses the Rab11-RE and the Rab4/Retromer routes to recycle back to the apical membrane. EGFR is required for the organisation of Serp/Crb endosomes, promoting the recycling of both cargoes to their final destination ensuring proper tube elongation.

In *crb* mutants apico-basal polarity is strongly affected, but a rudimentary tracheal system is still formed [44]. We analysed Serp accumulation in *crb* null mutants. *crb* mutants still deposit chitin (Fig 7A yellow arrow), indicating the capacity of these cells to organise in a tube, to localise apically the machinery for chitin deposition, and to secrete material into a luminal compartment. Serp accumulation in the luminal spaces was largely decreased or absent, and only occasionally we found Serp colocalising with CBP (Fig 7A blue arrow). Furthermore, when we overexpressed *crb* in the tracheal tissue we detected the expected apicalisation of the membranes, with accumulation of CBP around the cells and the formation of an abnormal lumen compartment. This indicates that all the machinery necessary to produce chitin is able to localise at the membrane, even if it is mislocalised. Strikingly, Serp was completely absent in the lumen of these embryos (Fig 7B). These results suggest that Crb regulates the accumulation of Serp in a specific manner.

In *serp* mutants Crb is found in apparently normal endosomes at stage 13–14. At later stages Crb is also found enriched in the SAR, however, this enrichment is slightly less conspicous as compared to the sibling control embryos (Fig 7C and 7D). This result suggested a possible role for *serp* in Crb recycling. To further explore this possibility we analysed Crb accumulation in the absence of *serp* in sensitised conditions, in particular in EGFR downregulation conditions. We found a strong phenotype, with the presence of large Crb-containing endosomes at early and late stages (st 14 and 16) (Fig 7E and 7F yellow arrows). Enrichment of Crb in the apical domain was hardly detected at late stages (Fig 7F blue arrowhead). These results raise the possibility that Serp plays a direct or indirect role in endocytic trafficking affecting Crb recycling.

In summary, our results would be consistent with the hypothesis that Serp and Crb are endocytic cargoes that in turn also regulate (directly or indirectly) the endocytic trafficking.

## Discussion

In this work we identify a novel role for EGFR in the regulation of the length of the tracheal tubes. Downregulation of EGFR leads to the overelongation of the main tracheal trunk. We find that EGFR regulates the accumulation and subcellular localisation of different factors known to participate in the control of tube length, in particular the apical determinant Crb and the chitin deacetylase Serp. Our results show that these two proteins traffic together in common sorting endosomes. EGFR affects the organisation of these Crb/Serp endosomes, likely impairing the correct delivery of both cargoes to their final destination. As a consequence, tube length is abnormally attained. Our results also show that these two cargoes are partitioned into different discrete domains within this common sorting endosome, consistent with the hypothesis that they use different retrieval pathways to recycle. In agreement with this it was shown that Serp undergoes retrograde transport using the Rab9-Retromer complex machinery to recycle to the lumen [36]. Here we find that, in the trachea, Crb also undergoes a complex pattern of recycling. Crb is first internalised in a temporal regulated manner. After internalisation part of the Crb pool is retrieved from the degradation pathway and would undertake different recycling routes (i.e the Rab4/Retromer short loop and the Rab11-RE route) to return to the membrane. This recycling pattern correlates with a temporally regulated localisation of Crb in different subcellular domains in the apical membrane: before and during internalisation Crb is high in the AFR, while internalisation promotes an enrichment of Crb the SAR (Model Fig 7G). Hence, our work identifies EGFR signalling as a hub to coordinate both cell intrinsic properties (Crb-mediated apical membrane growth) and extrinsic mechanisms (Serp modification of the aECM) that regulate tube elongation. We suggest that the regulation of the endocytic traffic of specific cargoes could be one of the molecular mechanisms downstream of the EGFR signal, and therefore could regulate different morphogenetic and pathological EGFR-mediated events.

### A role for EGFR in endocytic traffic regulation

We find that EGFR itself can also localise in the endosomes containing Crb and Serp. It has been already shown that upon activation, EGFR is internalised and that its activity is regulated by endocytic traffic. The endocytic trafficking of EGFR ensures, on the one hand, the downregulation of the signal by targeting the receptor to degradation, or its recycling back to the plasma membrane. The decision between being degraded or recycled depends, in turn, on signalling events targeting EGFR itself (for reviews see [45,46]). But besides being a cargo, the endocytic trafficking of EGFR can also serve as a platform for signalling. As such, EGFR could activate in the endosome downstream signalling cascades that reach the nucleus and regulate transcription (e.g. the MAPK pathway), or it could approach EGFR signalling to particular downstream effectors, residing in specific cellular organelles, regulating cytoplasmic targets [47,48].

Our results are consistent with a model where EGFR would activate signalling and regulate cytoplasmic targets, perhaps through its main signalling cascade, the ERK/MAPKinase pathway. EGFR signalling targets could be in the endosome itself [49]. Actually, it was shown that EGFR can regulate Rab5 activity through the regulation of Rab5 GAP and GEF proteins [50,51], and that ERK can regulate plasma membrane clathrin-independent recycling [52]. In addition, EGFR can phosphorylate Clathrin in a Src-mediated mechanism regulating its redistribution [53], can also phosphorylate the endocytic adaptor protein Eps15 [54,55], and can induce E-Cadh macropinocytosis [56]. Interestingly, myopic was shown to regulate EGFR signalling [57] and to interact with Rab4 [58]. Furthermore, Rac1, which can be a target of Receptor Tyrosine Kinases [59], has been shown to regulate Serp and Crb in the trachea [60]. In summary, while EGFR has been extensively analysed as an endocytic cargo, some reports also point to more direct effects of EGFR signalling upon the endocytic machinery, and therefore our analysis identifies a good system to further explore this mechanism. More work is required to identify the target of EGFR regulating endosomal trafficking in the trachea, however, our results suggest that EGFR could directly or indirectly target WASH-promoted actin organisation or the Retromer complex, which is required for WASH recruitment.

### Tube elongation

The elongation of the tracheal tubes depends largely on the proper organisation of an aECM, which acts as a viscoelastic matrix that restricts excessive elongation [7,14,15]. On the other hand, Crb-dependant apical membrane growth [7,11] and pSrc-regulated cell shape changes [12,13] also control tube length. How the aECM instructs the underlying tracheal epithelium to adjust its apical enlargement is not fully understood, although mechanical or signalling events, or a combination of both, may be required. Nevertheless, it is likely that the cell intrinsic mechanisms (i.e. apical membrane expansion and/or cell shape changes) and cell extrinsic mechanisms (i.e. aECM) are coordinated rather than being two independent mechanisms controlling the same morphogenetic event. Indeed, some reports propose that the aECM may be anchored to the apical cell membrane through connecting proteins such as ZP proteins [7,8,61–63]. In such case, it still remains to be explained if these proteins transduce the signal to regulate Crb-mediated apical membrane growth.

Our observation that one single signalling pathway, EGFR, controls both intrinsic and extrinsic tube elongation mechanisms provides an explanation to further understand the coordination of these mechanisms. Furthermore, our results suggest that the trafficking of Crb and Serp may influence each other, adding an extra layer of coordination to the system. Actually, a role for Crb in regulating endocytic trafficking has been already proposed [64]. Indeed, several reports indicate that polarity proteins are both cargoes and regulators of endocytic recycling [65]. Crb seems to control Serp trafficking in the trachea, and accordingly we find that when overexpressed, Serp is lost from the lumen, maybe because the excess of Crb saturates all Retromer complex precluding Serp recycling. In turn, Serp can also influence Crb trafficking, particularly in sensitised backgrounds. While Serp has been proposed to act as a chitin deacetylase [14,15], it cannot be ruled out that it also plays a role in endocytic trafficking. Further experiments are required to determine whether Crb and Serp exert a direct instructive role by controlling specific endocytic regulator/s, or an indirect one by altering the trafficking homeostasis, by competing for common trafficking factors or by stimulating compensatory trafficking pathways.

The interdependence of different tube length regulators may mask the exact contribution of each one to tube enlargement. Hence, a clear correlation of tube size with excess or absence of different tube length regulators may be difficult to be established. In agreement with this it has been shown that both the absence of Serp and its overexpression gives rise to extralong tubes [15].

### The regulation of Crb localisation in the trachea

Our work provides insights into Crb accumulation during morphogenesis and the role of recycling in the process. Crb requires the Retromer complex for recycling [41,42]. The Retromer complex is typically implicated in the retrieval of cargoes from endosomes to TGN via the retrograde pathway [33,35]. However, it was proposed that Rab9, required for TGN trafficking in the trachea, did not affect Crb accumulation [36]. Furthermore, in HeLa cells Crb2 undergoes a rapid recycling to the membrane without accumulating in the TGN [41]. Besides this role of Retromer in retrograde transport, a Retromer-mediated traffic directly from endosomes to the plasma membrane was also identified [43]. Rab4, which was known to participate in a rapid recycling pathway back to the membrane [30], associates with Retromer tubules for this direct traffic [43]. As Crb requires Retromer, does not undertake the retrograde pathway and is found in Rab4 endosomes, we propose that it undergoes a rapid direct recycling to the apical membrane regulated by Rab4/Retromer.

Both Serp and Crb require the Retromer complex for recycling, but they seem to undergo different Retromer-dependent pathways. How does the complex select the cargo? Retromer interacting proteins, like sorting nexins, might provide cargo specificity loading distinct transport intermediates, which nevertheless use the same core machinery for membrane budding and fission [32–35]. Interestingly, the nexin Snx27 has been shown to participate in the direct Retromer pathway to the plasma membrane. Snx27 is unique because it contains a PDZ domain that binds to PDZ-binding motifs (PDZbm) in cargo proteins [43,66]. Because Crb contains a PDZbm [67] and it is recycled through the retromer complex [41,42], we speculated that Snx27 could be involved in this process. We find that CG32758 is the ortholog of Snx27 in *Drosophila*. While more experiments are required to draw definitive conclusions, our preliminary data point to the participation of Snx27-CG32758 in Crb trafficking to plasma membrane, likely in a Rab4/Retromer dependent pathway.

Finally, our results indicate that during tracheal development Crb can concentrate in different subdomains of the apical membrane. At early stages it is strongly concentrated in the AFR, in direct contact with the lumen, and later it becomes enriched preferentially in the SAR. Accumulation of Crb in the SAR or more diffusely in the AFR has also been observed in other *Drosophila* ectodermal tissues [68] or in other organisms, as it is the case of mammalian Crb3 that is present both on the apical surface and at Tight Junctions [69]. Whether the specific subcellular apical localisation of Crb performs different functions (e. g. in the case of the trachea, SAR accumulation directing tube elongation while AFR accumulation maintaining apicobasal polarity) is an interesting issue that remains to be investigated. We find that the late enrichment of Crb in the SAR depends on Rab5-mediated endocytosis, as when it is blocked Crb remains high in all apical domain. Crb is internalised and accumulates in endosomes, which are abundant preceding SAR enrichment, from where a fraction of the internalised protein follows the degradation pathway and another fraction recycles back to the apical region. Crb apical recycling could depend on two different endocytic itineraries: A Rab4/Retromer pathway could recycle Crb rapidly to the apical membrane, preferentially to the AFR, while a longer loop involving Rab11-RE would also recycle Crb apically, preferentially to the SAR. Alternatively, our results are also consistent with a temporally regulated model, in which right after internalisation (stages 13 and 14) Crb is recycled by any of the two pathways preferentially to the AFR. As Rab4 involves a shorter loop it could be more relevant in this phase. In a later cellular context (from stage 15) the recycling is preferentially routed to the SAR. The involvement of these two recycling pathways, or different recycling phases, in Crb sorting may be a general mechanism. Actually, when endocytosis is prevented in 3D-MDCK cells Crb3 levels are increased and it is not able to relocalise from the apical domain to the Tight Junctions [70]. Understanding the molecular mechanisms regulating Crb accumulation, including its recycling, is a key question given the importance of Crb in organisms.

## Materials and methods

### Drosophila strains

The following stocks are described in Flybase: *y* ^*1*^*w* ^*118*^ (wild type, WT), *UAS-Egfr*^*DN*^, *UAS-Egfr*^*CA*^ (*UAS-Egfr*^*λtop*^), *UAS-btl*^*DN*^, *UAS-btl*^*CA*^ (*UAS-λbtl*), *UAS-Rab5*^*DN*^ (*UAS-Rab5*^*S43N*^), *UAS-Rab5*^*CA*^ (*UAS-Rab5*^*Q88L*^), *UAS-Rab4*^*DN*^*-YFP* (*UAS-Rab4*^*S22N*^*-YFP*), *UAS-Rab4*^*CA*^ (*UAS-RAB4*^*Q67L*^), *Rab4*^*EYFP*^, *UAS-Rab11*^*DN*^*-YFP* (*UAS-Rab4*^*S25N*^*-YFP*), *UAS-Crb*^*FL*^ (gift from E. Knust), *Crb*^*11A22*^. *serp*^*RB*^ mutants and *UAS-Serp-CBD-GFP* were kindly provided by S. Luschnig. Knock in alleles *Crb*^*GFP*^*-c* and *Crb*^*Cherry*^ were kindly provided by Y. Hong.

For overexpression in the trachea from invagination onwards we used *btlGal4* or *btlGal4-UAS-Src-GFP* (to mark tracheal cells with Src membrane protein). *AbdBGal4* was used to drive expression in the posterior part of the embryo.

### Immunofluorescent stainings and dextran injections

Immunostainings were performed on embryos fixed in 4% formaldehyde for 20 minutes, except for E-Cadh stainings, for which embryos were fixed for 10 minutes. The following primary antibodies were used: mouse anti-EGFR Extracellular domain (1:500) from Sigma; mouse anti-Crb (Cq4) (1:20), rat anti-E-Cadh (DCAD2) 1:100 and mouse anti-Wash P3 (1:50) from Developmental Studies Hybridoma Bank, DSHB; rabbit anti-Serp (1:300) generously provided by S. Luschnig; rabbit anti-Rab11 (1:2000) and guinea pig anti-Rab5 (1:1000) were generously provided by T. Tanaka; guinea-pig anti-Uif (1:500) generously provided by R. Ward; rabbit or goat anti-GFP (1:600) from Molecular Probes and Roche used to visualise both GFP and YFP-tagged proteins; chicken anti-ßgal (1:300) from Abcam. Alexa Fluor 488, 555, 647 (Invitrogen) or Cy2-, Cy3-, Cy5-Conjugated secondary antibodies (Jackson ImmunoResearch) were used at 1:300 in PBT 0.5% BSA. CBP (Chitin Binding Protein) was visualised as a secondary antibody at 1:300. Rhodamine-labelled 10 KDa dextran injections were used for an in vivo endocytosis assay and were performed as described in [4].

### Image acquisition

Fluorescence confocal images of fixed embryos were obtained with a Leica TCS-SPE system. Unless otherwise indicated, images shown are maximum projections of Z stack sections (0.2–0.4 μm). Images were imported into Fiji and Photoshop, and assembled into figures using Illustrator.

### Time lapse movies

Dechorionated embryos were mounted and lined up on a Menzel-Gläser cover slips with oil 10-S Voltalef (VWR) and covered with a membrane (YSI membrane kit). Life imaging was performed on a Zeiss Lsm780 Confocal and Multiphoton System. For Movie 1 a 950 nm Multiphoton laser MaiTai HP DS was used. For movies 2–4 a 488 /514 nm Argon laser was used. In all movies we used an oil 63x/1.4 NA objective. To visualize time-lapse movies, maximal intensity projections are shown.

### Morphometric analyses

#### Tube size

Confocal projections were used to analyse the length of the embryonic dorsal trunk (DT) stained with CBP in stage 16 embryos. We traced two paths using the freehand line selection tool of Fiji software between the junction DT/Transverse Connective (TC) from metamere 4 to 9, one following DT curvature and a straight one. DT length was expressed as the ratio between the real path and the straight line. A ratio of 1 reflects a straight DT.

#### Cell junction lengths

Cell junctions were classified according to the angles (tube axis set to 0°), considering axial junctions those oriented 0°±30° and circumferential junctions 90°±30°. The freehand line selection tool of Fiji software was used to measure the length of the junctions.

#### Crb intensity levels

Projections of confocal sections that include MT and a piece of DT were used for the analysis. *EGFR*^*DN*^*/EGFR*^*CA*^ transgenes were expressed in the tracheal cells by means of *btlGal4* but not in the MT. We measured the levels in pixels in each structure using the freehand line selection tool of Fiji, tracing a line including the whole tube (line with 60 for DT and line with 10 for MT). Fluorescence intensity of Crb in the DT was normalised to the Crb levels in MT in each embryo. We compared the ratio of Crb levels in the trachea/MT among the experimental conditions and the internal control wild type embryos.

#### Crb subcellular accumulation

We quantified the total levels of Crb (using the Sum Fluorescent Intensity in Fiji) in different apical subcellular domains. We selected individual cells from the region of the DT between metameres 7 and 9, and generated projections of few sections to include only the whole cell or a small number of them. We quantified Crb in SAR by outlining the cell contour (using E-Cadh to visualise it) and Crb in AFR by drawing a freehand section inside. We express the subcellular accumulation as the ratio between SAR/AFR.

#### Crb Serp colocalization

Colocalisation analysis was performed using the ImageJ plugin Colocalisation highlighter, considering colocalisation when the ratio of fluorescence intensities between red and green channels was above 0.5. Those fluorescence intensities above the threshold appear in a binary image colour as white (colocalised points). From this mask, we selected manually each colocalised vesicle by wand tool in Fiji and added it in the ROI Manager. Thereafter, to analyse the colocalisation, a ratio between the number of pixels (integrated density) of one channel that colocalises with the marker in the other channel and the total number of pixels above the threshold measured for each channel was measured.

#### Analysis of cargoes/endosome

For each region of DT analysed, we counted in each stack the number of endosomes containing both Crb and Serp, Crb alone or Serp alone. We analysed the proportion of each type of vesicle.

#### Cell number

To count cell number, embryos were stained with anti-Uif, anti-laminin A and DAPI to label apical and basement membrane, and the nuclei of tracheal cells. To analyse the cell number, we counted in each stack the nuclei from the region of the DT between metameres 7 and 8 in stage 15–16 embryos. Images were obtained with Leica DMI6000 TCS-SP5 laser confocal microscope 63x/1.4 NA oil using 405 nm diode laser.

### Quantifications and statistics

Total number of cells/embryos is provided in text and figures. Error bars indicate standard error (s.e.). *P*-values were obtained with an unpaired two-tailed Student's *t*-test using STATA 12.1 software. *P<0.05, 0.001<**P<0.01, ***P<0.001.

## Supporting information

S1 Fig Quantification of tube size. (A-C) Lateral views of stage 16 embryos stained for GFP (green) and CBP (red) to visualise the lumen. Compare the elongated DT when EGFR is downregulated (*btlGal4-UASsrcGFP-UASEGFR*^*DN*^) to control (*UAS-EGFR*^*DN*^) and to EGFR overactivation (*btlGal4-UASsrcGFP-UASEGFR*^*CA*^). Scale bar 50 μm. (D-G) Quantification of the length of the Dorsal Branch (DB) and the Transverse Connective (TC) of metamere 5 (D and E respectively), the Dorsal Trunk (DT) diameter measured in the region between tracheal metameres 7 and 8 (F) and the total length of the embryo measured from the most anterior to most posterior region (G). The measures are shown in (A). Note that only the diameter of the DT of EGFR^CA^ mutants is significantly different from the control, with a P<0.05 by Student's t-test. n refers to the number of embryos analysed. (H-K) Effects of Btl activity modulation. (H,J) Lateral views of stage 16 embryos stained for GFP (green) and CBP (red) to visualise the lumen. (I,K) Lateral views showing 2 tracheal metameres of stage 16 embryos stained with E-Cadh to visualise apical cell shape. The downregulation (*btlGal4-UASsrcGFP-UASbtl*^*DN*^) or the constitutive activation of *btl* (*btlGal4-UASsrcGFP-UASbtl*^*CA*^) does not give rise to tube elongation or cell shape defects. In contrast, defects of lack of terminal branching and fusion are detected when *btl* is downregulated (arrows in H) and excess of terminal branching and missguidances are detected when *btl* is constitutively activated (arrows in J). Scale bar H 50 μm, I 10 μm.
(TIF)

S2 Fig Accumulation of Serp in EGFR^CA^. (A-F) Lateral views of control embryos and embryos carrying *btlGal4-UAS-EGFR*^*CA*^ at the indicated stages. Embryos are stained with Serp antibody. Serp is detected in the lumen (pink arrows) and in the apical membrane of tracheal cells (blue arrows). When EGFR is constitutively activated high levels of Serp are detected in the lumen. Scale bars A,F 10 μm, B-E 25 μm.
(TIF)

S3 Fig Accumulation of Crb in EGFR mutants. (A-L) Lateral views of representative stage 16 control embryos or embryos expressing the indicated transgenes in the trachea (using *btlGal4*) stained for Crb. Panels include the DT of 3 tracheal metameres and a MT to compare Crb levels. Note that MT do not express EGFR constructs. Scale bar 25 μm. (M-O) Details of 1 single tracheal metamere of stage 16 embryos of the indicated genotypes stained for E-Cadh (green, white) to visualise the apical domain and Crb (red, white). Scale bar 7,5 μm.
(TIF)

S4 Fig Accumulation of Crb in Rab mutants. (A,F) Lateral views of embryos of the indicated genotypes stained for Crb (red, white) and E-Cadh (green, white) or CBP (green, white) showing 1–2 tracheal metamere. Stages are indicated. (A) shows the last tracheal metamere, in contact with the spiracle. *btlGal4* expression limit is marked by a blue line. Note that while Crb vesicles are absent in the tracheal region, they are still detected in the spiracle (blue arrows). (A-D) Note that at early stages the pattern of Crb accumulation is similar to control when Rab11 or Rab4 are downregulated. (E,F) At late stages Crb is sharply accumulated in the SAR in Rab4^DN^ mutants. Scale bars A,E 10μm, B 5μm and F 7,5μm.
(TIF)

S5 Fig Accumulation of Crb in EGFR mutants. (A-H) Lateral views of representative stage 14 control embryos or embryos expressing EGFR^DN^ in the trachea stained for E-Cadh (green, white) and Crb (red, white). Note that in EGFR^DN^ mutants the enrichment of Crb in the SAR is more conspicuous at this stage as compared to control embryos. Scale bar 7,5 μm.
(TIF)

S6 Fig Accumulation of Crb, Serp and EGFR and EGFR requirement. (A-C') Lateral views of stage 14 WT embryos stained for Crb (red) and Serp (green). Each image in A,B,C corresponds to a single confocal stack. Panels marked with 1 and 2 correspond to the insets shown in A-C. Below, the same image is shown with a co-localisation point mask visualised in white. Note that many endosomes accumulate both Serp and Crb, but endosomes containing only Serp or Crb are also found. In endosomes containing both Crb and Serp, the two proteins are sorted into different endosomal domains, colocalising in a region (insets). Scale bar 5 μm. (D) Shows a scheme to represent the different type of vesicles found (Crb/Serp_e_, Serp_e_ and Crb_e_). Crb and Serp partially colocalise (white). (E,F) Lateral views of st 14 embryos expressing EGFR^DN^ in tracheal cells. Endosomes are different from control, and Serp (E) and Crb (F) accumulate abnormally. Scale bar E 2,5 μm, F 5 μm. (G,H) Lateral views of *Crb*^*Cherry*^ embryos at stage 16. The embryos show a normal tracheal pattern (visualised with CBP in G) and normal cell organisation (visualised with E-Cadh in H). Scale bar G 50 μm, H 10 μm. (I) Lateral view of a stage 14 embryo carrying *btlGal4*-*UASserp-CBD-GFP* stained for Serp (red, white) and GFP (green, white) showing 1 tracheal metamere. Note that Serp and GFP largely colocalise, indicating that Serp-CBD-GFP recapitulates Serp accumulation. Occasionally we find endosomes containing only endogenous Serp protein (red arrow in I) or Serp-CBD-GFP (green arrow in I; note that Serp antibody does not recognise Serp-CBD-GFP protein, [14]). Scale bar 5 μm. (J-L) Lateral views of stage 14 embryos stained for EGFR, Serp and/or GFP (in embryos carrying Crb^GFP^ in J,L). EGFR itself is found very often in Serp and/or Crb containing endosomes (white arrows). Scale bar J 5μm, K,L 2,5μm.
(TIF)

S7 Fig (A-C) Time-lapse images of stage 17 embryos carrying *btlGal4-UASSerp-CBD-GFP* injected with rhodamine-labelled dextran in the different genetic backgrounds indicated. Intracellular dextran punctae largely co localise with Serp-CBD-GFP containing endosomes, indicating internalisation. Note that dextran internalisation is detected in downregulation and constitutively active conditions for EGFR. Also note that Serp-CBD-GFP/Dextran containing endosomes are abnormally big compared with the control when EGFR is downregulated (pink arrows in B). Scale bar 5 μm.
(TIF)

S1 Video Crb apical subcellular accumulation. Embryo carrying *Crb*^*GFP*^ visualised from a lateral view using an inverted Zeiss Lsm780 confocal with 63x Oil objective and a 2 zoom. Images were taken every 2 minutes during 2,30 hours in 20 Z-stack of 0,5μm, from early stage 14 to stage 15. Note that at early stages Crb is detected in the AFR of DT cells (yelow arrow). As development proceeds Crb becomes enriched in the SAR (red arrows).
(AVI)

S2 Video Rab4 and Crb accumulation. Stage 14 embryo carrying *Rab4*^*EYFP*^ *and Crb*^*Cherry*^ visualised from a lateral view using an inverted Zeiss Lsm780 confocal with 63x Oil objective and a 2,5 zoom. Images were taken continuously in one single Z-stack during two minutes. Note that many Crb vesicles are also positive for Rab4 (white arrows).
(AVI)

S3 Video Serp and Crb accumulation. Stage 14 embryo carrying *btlGal4-UAS-Serp-CBD-GFP and Crb*^*Cherry*^ visualised from a lateral view using an inverted Zeiss Lsm780 confocal with 63x Oil objective and a 2,5 zoom. Images were taken continuously in a single Z-stack during 3,5 min. Crb and Serp are highly dynamic, and move around the cell.
(AVI)

S4 Video Serp and Crb accumulation. Stage 14 embryo carrying *btlGal4-UAS-Serp-CBD-GFP and Crb*^*Cherry*^ visualised from a lateral view using an inverted Zeiss Lsm780 confocal with 63x Oil objective and a 2,5 zoom. Images were taken continuously in a single Z-stack during 6 min. Crb and Serp are highly dynamic, and move around the cell.
(AVI)
