# Supplementary figures and images for: EGFR controls Drosophila tracheal tube elongation by intracellular trafficking regulation
Source: PLoS Genet. 2017 Jul 5;13(7):e1006882. doi: 10.1371/journal.pgen.1006882 (PMC5517075; doi:10.1371/journal.pgen.1006882)

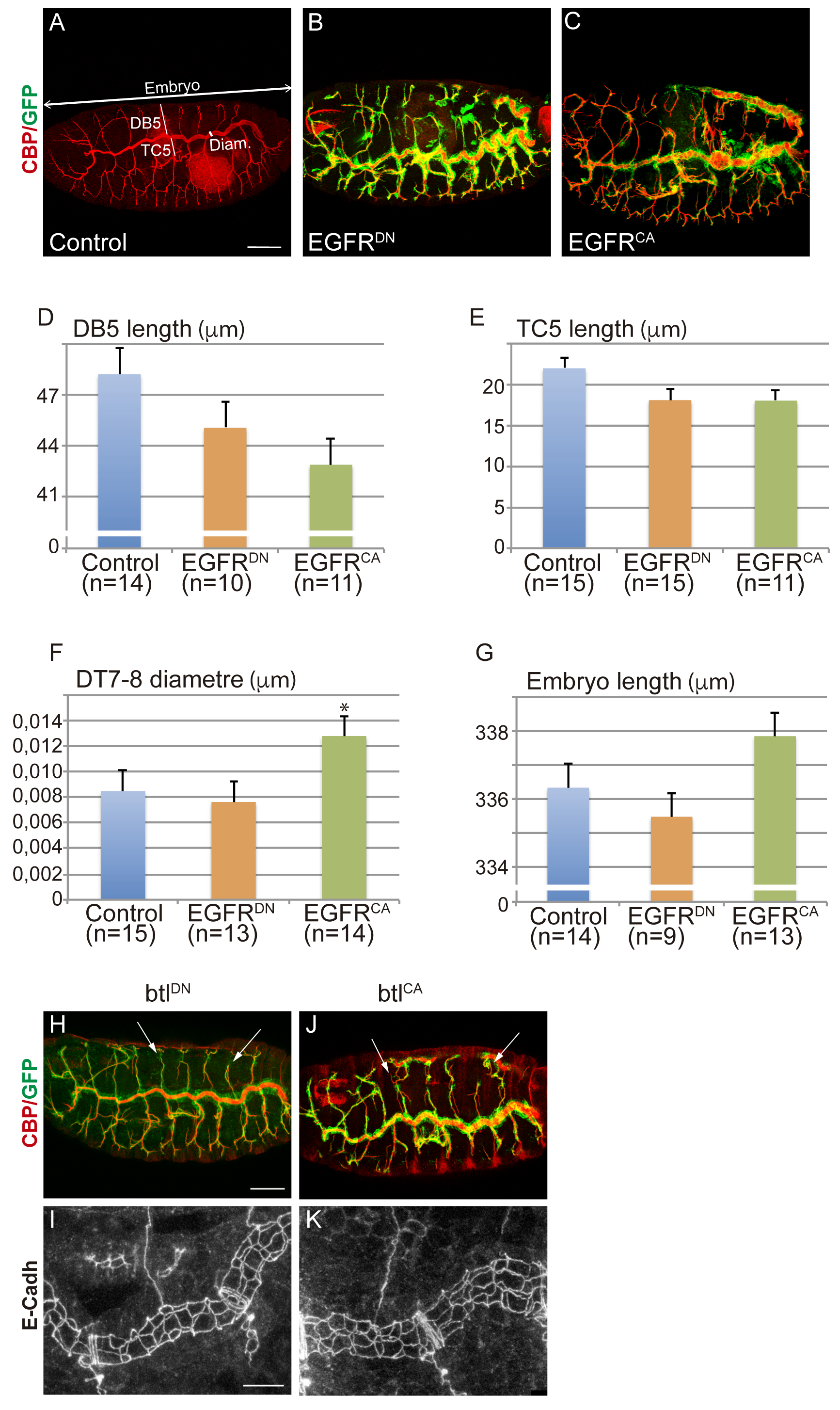

Supplement: S1 Fig — (A-C) Lateral views of stage 16 embryos stained for GFP (green) and CBP (red) to visualise the lumen. Compare the elongated DT when EGFR is downregulated (btlGal4-UASsrcGFP-UASEGFRDN) to control (UAS-EGFRDN) and to EGFR overactivation (btlGal4-UASsrcGFP-UASEGFRCA). Scale bar 50 μm. (D-G) Quantification of the length of the Dorsal Branch (DB) and the Transverse Connective (TC) of metamere 5 (D and E respectively), the Dorsal Trunk (DT) diameter measured in the region between tracheal metameres 7 and 8 (F) and the total length of the embryo measured from the most anterior to most posterior region (G). The measures are shown in (A). Note that only the diameter of the DT of EGFRCA mutants is significantly different from the control, with a P<0.05 by Student's t-test. n refers to the number of embryos analysed. (H-K) Effects of Btl activity modulation. (H,J) Lateral views of stage 16 embryos stained for GFP (green) and CBP (red) to visualise the lumen. (I,K) Lateral views showing 2 tracheal metameres of stage 16 embryos stained with E-Cadh to visualise apical cell shape. The downregulation (btlGal4-UASsrcGFP-UASbtlDN) or the constitutive activation of btl (btlGal4-UASsrcGFP-UASbtlCA) does not give rise to tube elongation or cell shape defects. In contrast, defects of lack of terminal branching and fusion are detected when btl is downregulated (arrows in H) and excess of terminal branching and missguidances are detected when btl is constitutively activated (arrows in J). Scale bar H 50 μm, I 10 μm. (TIF) [file pgen.1006882.s001.tif]

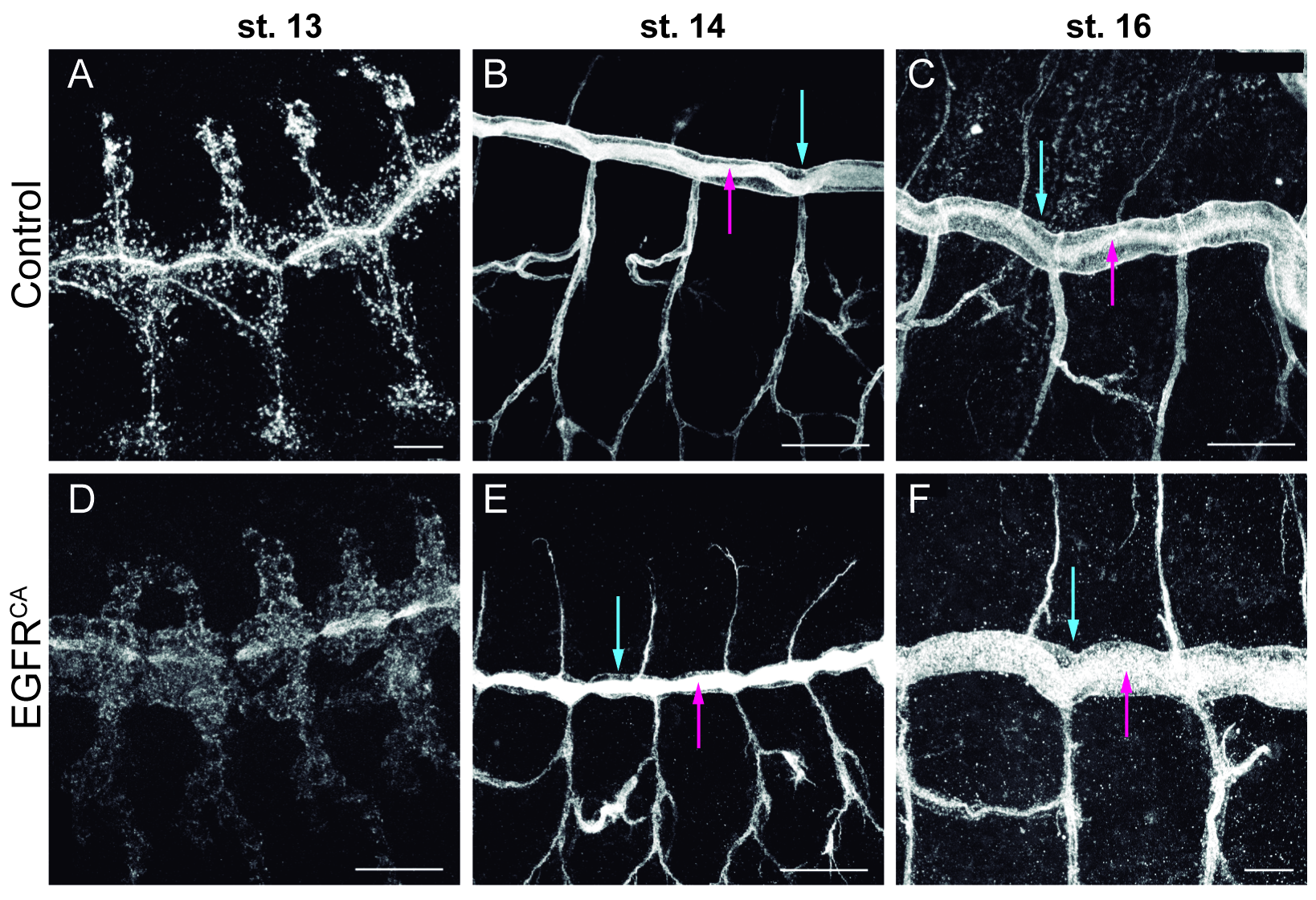

Supplement: S2 Fig — (A-F) Lateral views of control embryos and embryos carrying btlGal4-UAS-EGFRCA at the indicated stages. Embryos are stained with Serp antibody. Serp is detected in the lumen (pink arrows) and in the apical membrane of tracheal cells (blue arrows). When EGFR is constitutively activated high levels of Serp are detected in the lumen. Scale bars A,F 10 μm, B-E 25 μm. (TIF) [file pgen.1006882.s002.tif]

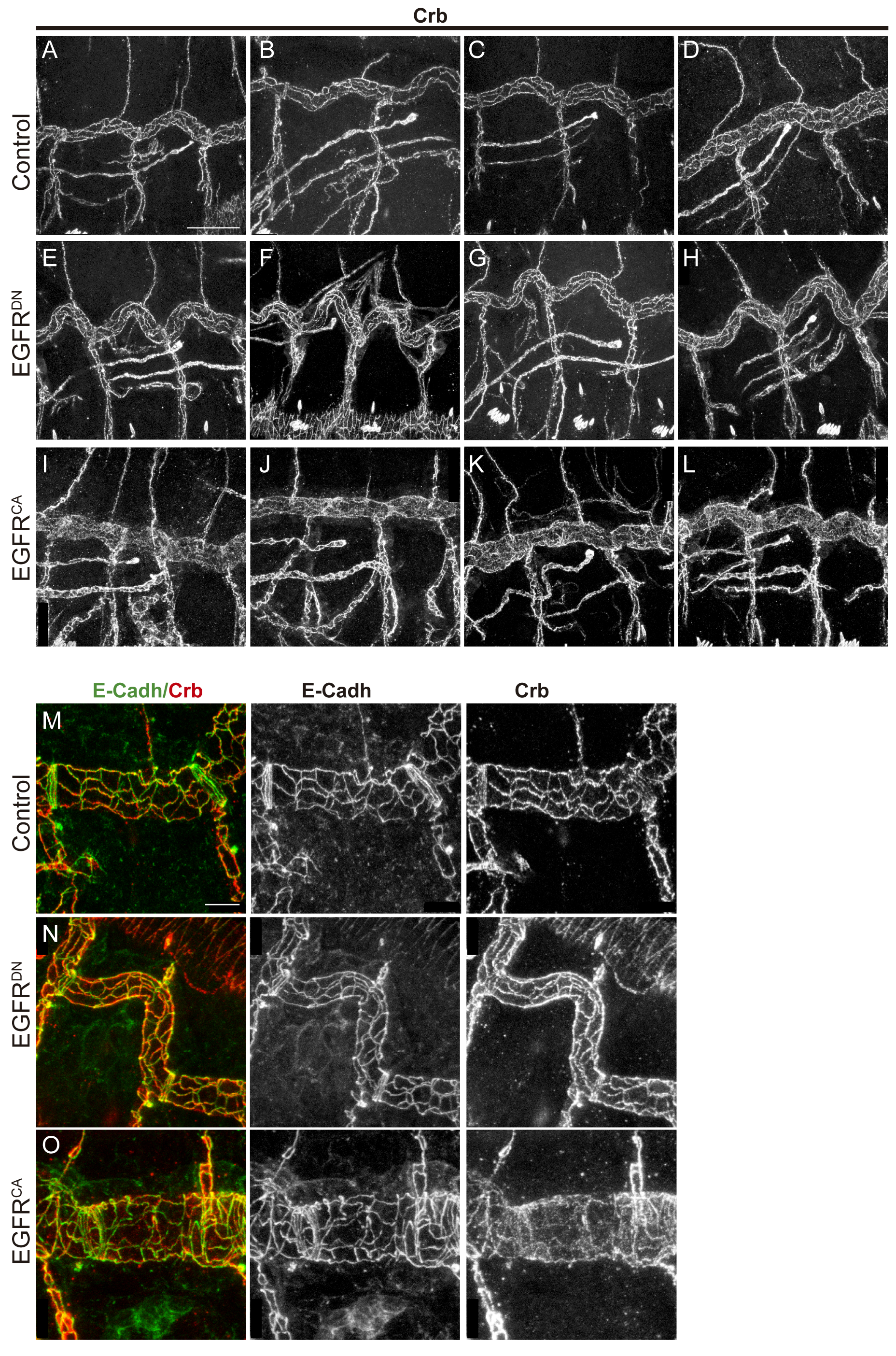

Supplement: S3 Fig — (A-L) Lateral views of representative stage 16 control embryos or embryos expressing the indicated transgenes in the trachea (using btlGal4) stained for Crb. Panels include the DT of 3 tracheal metameres and a MT to compare Crb levels. Note that MT do not express EGFR constructs. Scale bar 25 μm. (M-O) Details of 1 single tracheal metamere of stage 16 embryos of the indicated genotypes stained for E-Cadh (green, white) to visualise the apical domain and Crb (red, white). Scale bar 7,5 μm. (TIF) [file pgen.1006882.s003.tif]

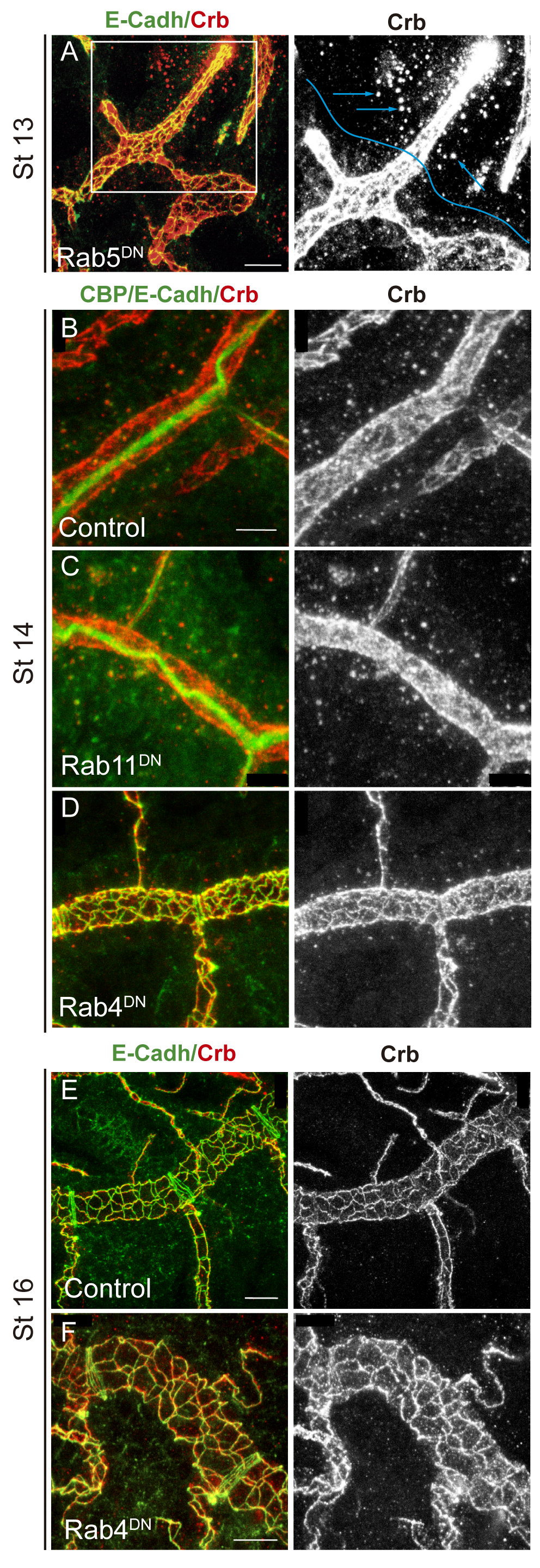

Supplement: S4 Fig — (A,F) Lateral views of embryos of the indicated genotypes stained for Crb (red, white) and E-Cadh (green, white) or CBP (green, white) showing 1–2 tracheal metamere. Stages are indicated. (A) shows the last tracheal metamere, in contact with the spiracle. btlGal4 expression limit is marked by a blue line. Note that while Crb vesicles are absent in the tracheal region, they are still detected in the spiracle (blue arrows). (A-D) Note that at early stages the pattern of Crb accumulation is similar to control when Rab11 or Rab4 are downregulated. (E,F) At late stages Crb is sharply accumulated in the SAR in Rab4DN mutants. Scale bars A,E 10μm, B 5μm and F 7,5μm. (TIF) [file pgen.1006882.s004.tif]

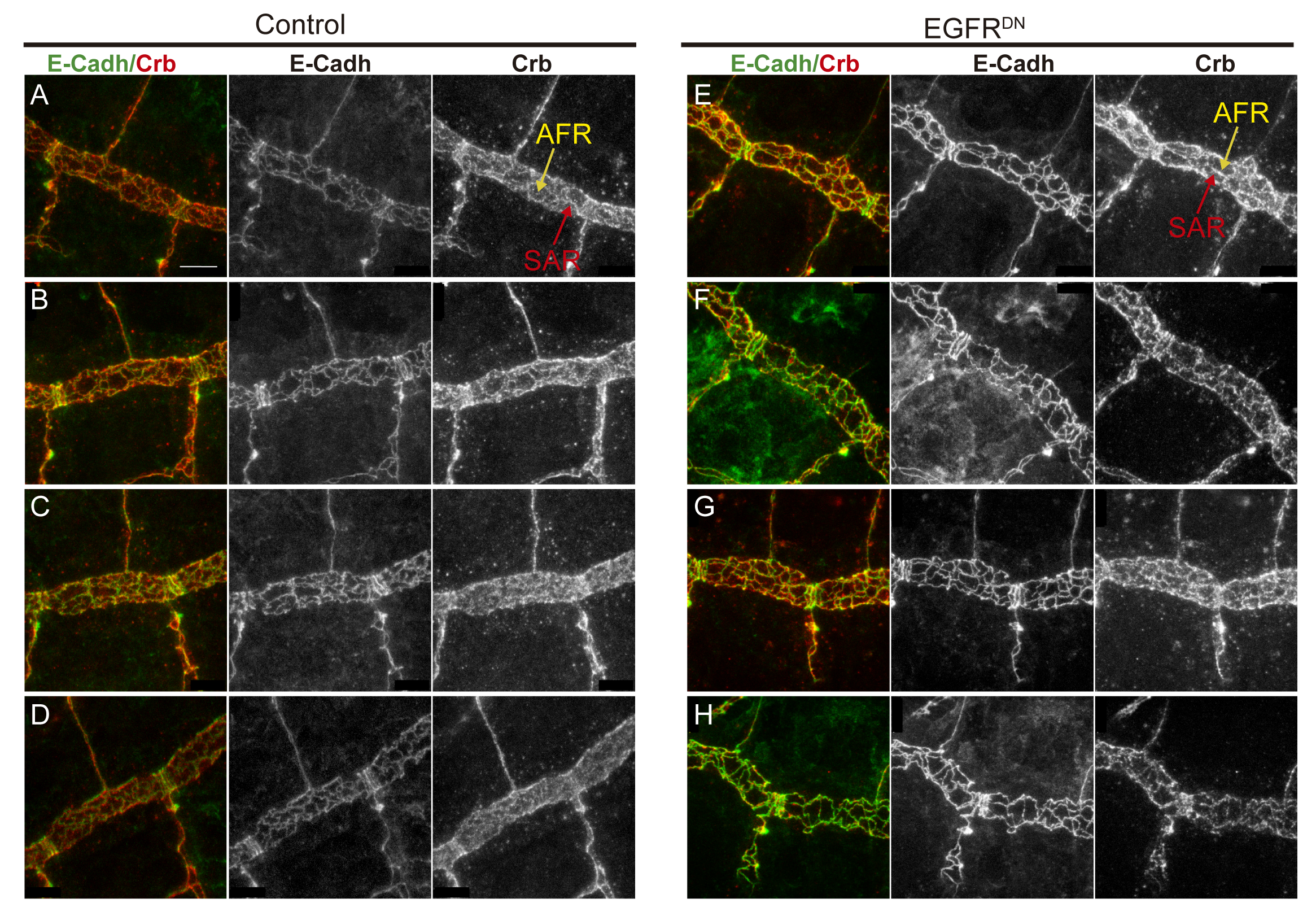

Supplement: S5 Fig — (A-H) Lateral views of representative stage 14 control embryos or embryos expressing EGFRDN in the trachea stained for E-Cadh (green, white) and Crb (red, white). Note that in EGFRDN mutants the enrichment of Crb in the SAR is more conspicuous at this stage as compared to control embryos. Scale bar 7,5 μm. (TIF) [file pgen.1006882.s005.tif]

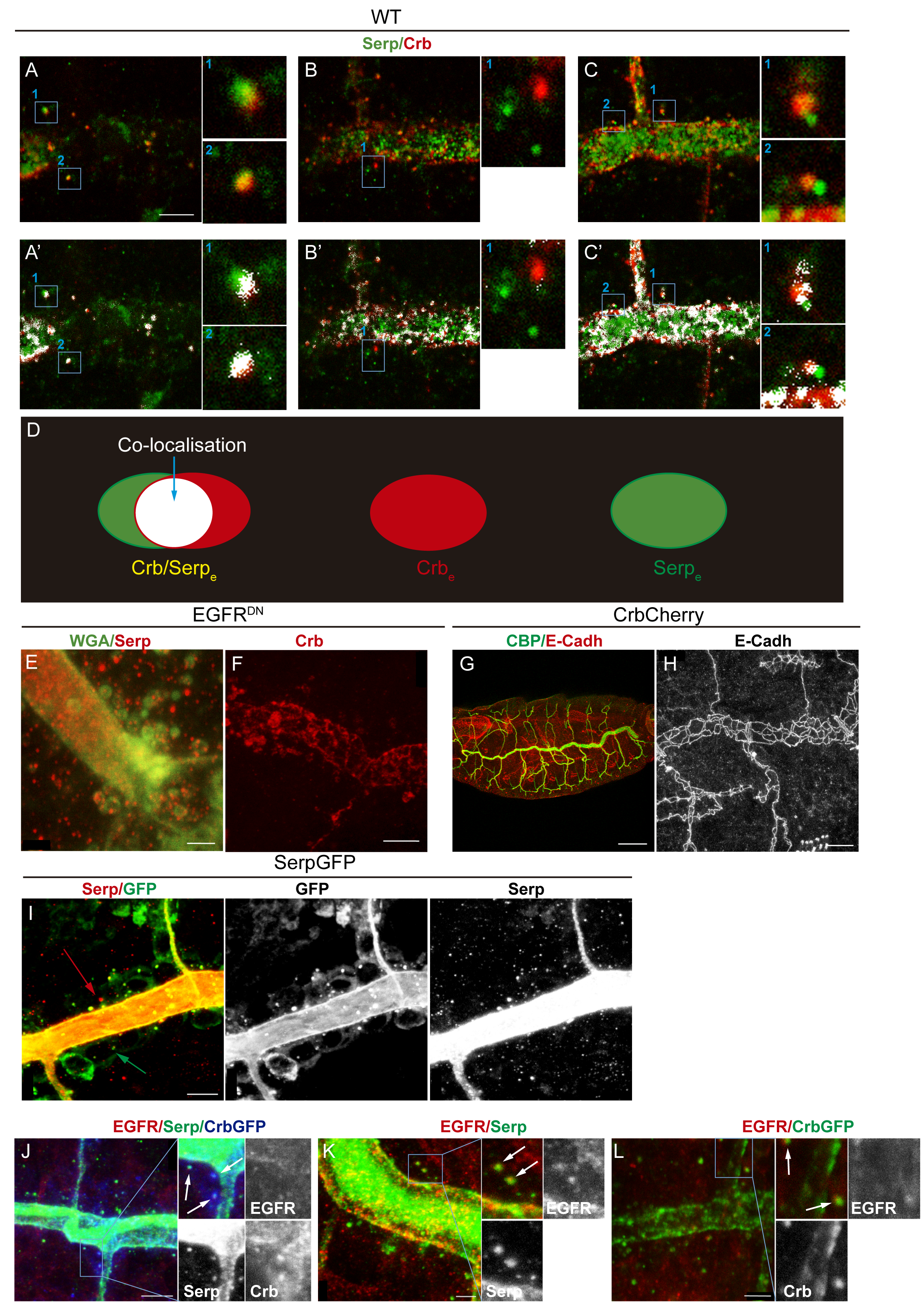

Supplement: S6 Fig — (A-C') Lateral views of stage 14 WT embryos stained for Crb (red) and Serp (green). Each image in A,B,C corresponds to a single confocal stack. Panels marked with 1 and 2 correspond to the insets shown in A-C. Below, the same image is shown with a co-localisation point mask visualised in white. Note that many endosomes accumulate both Serp and Crb, but endosomes containing only Serp or Crb are also found. In endosomes containing both Crb and Serp, the two proteins are sorted into different endosomal domains, colocalising in a region (insets). Scale bar 5 μm. (D) Shows a scheme to represent the different type of vesicles found (Crb/Serpe, Serpe and Crbe). Crb and Serp partially colocalise (white). (E,F) Lateral views of st 14 embryos expressing EGFRDN in tracheal cells. Endosomes are different from control, and Serp (E) and Crb (F) accumulate abnormally. Scale bar E 2,5 μm, F 5 μm. (G,H) Lateral views of CrbCherry embryos at stage 16. The embryos show a normal tracheal pattern (visualised with CBP in G) and normal cell organisation (visualised with E-Cadh in H). Scale bar G 50 μm, H 10 μm. (I) Lateral view of a stage 14 embryo carrying btlGal4-UASserp-CBD-GFP stained for Serp (red, white) and GFP (green, white) showing 1 tracheal metamere. Note that Serp and GFP largely colocalise, indicating that Serp-CBD-GFP recapitulates Serp accumulation. Occasionally we find endosomes containing only endogenous Serp protein (red arrow in I) or Serp-CBD-GFP (green arrow in I; note that Serp antibody does not recognise Serp-CBD-GFP protein, [14]). Scale bar 5 μm. (J-L) Lateral views of stage 14 embryos stained for EGFR, Serp and/or GFP (in embryos carrying CrbGFP in J,L). EGFR itself is found very often in Serp and/or Crb containing endosomes (white arrows). Scale bar J 5μm, K,L 2,5μm. (TIF) [file pgen.1006882.s006.tif]

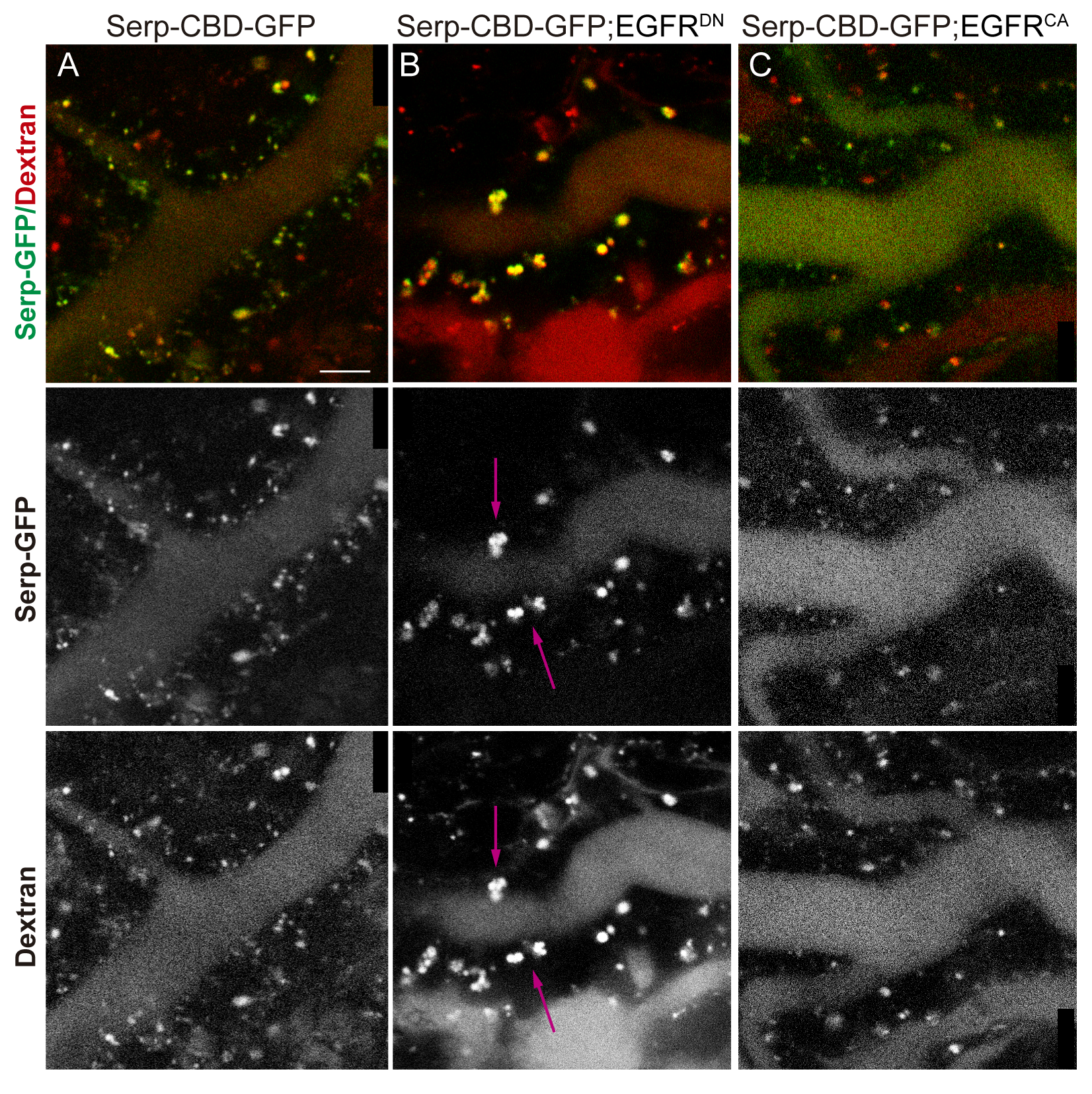

Supplement: S7 Fig — Intracellular dextran punctae largely co localise with Serp-CBD-GFP containing endosomes, indicating internalisation. Note that dextran internalisation is detected in downregulation and constitutively active conditions for EGFR. Also note that Serp-CBD-GFP/Dextran containing endosomes are abnormally big compared with the control when EGFR is downregulated (pink arrows in B). Scale bar 5 μm. (TIF) [file pgen.1006882.s007.tif]
